# Anticancer Activity of Natural Compounds from Plant and Marine Environment

**DOI:** 10.3390/ijms19113533

**Published:** 2018-11-09

**Authors:** Anna Lichota, Krzysztof Gwozdzinski

**Affiliations:** Department of Molecular Biophysics, Faculty of Biology and Environmental Protection, University of Lodz, 90-136 Lodz, Poland; anna.lichota@biol.uni.lodz.pl

**Keywords:** natural compounds, anticancer properties, substances from marin

## Abstract

This paper describes the substances of plant and marine origin that have anticancer properties. The chemical structure of the molecules of these substances, their properties, mechanisms of action, their structure–activity relationships, along with their anticancer properties and their potential as chemotherapeutic drugs are discussed in this paper. This paper presents natural substances from plants, animals, and their aquatic environments. These substances include the vinca alkaloids, mistletoe plant extracts, podophyllotoxin derivatives, taxanes, camptothecin, combretastatin, and others including geniposide, colchicine, artesunate, homoharringtonine, salvicine, ellipticine, roscovitine, maytanasin, tapsigargin, and bruceantin. Compounds (psammaplin, didemnin, dolastin, ecteinascidin, and halichondrin) isolated from the marine plants and animals such as microalgae, cyanobacteria, heterotrophic bacteria, invertebrates (e.g., sponges, tunicates, and soft corals) as well as certain other substances that have been tested on cells and experimental animals and used in human chemotherapy.

## 1. Introduction

The development of cancer registries throughout the world has led to a search for novel drugs that are toxic to the cancer cells while having no harmful effect on normal cells. The anticancer drugs used previously exhibited relatively high toxicity not only to the tumour cells, but also to the normal cells of the body part in which the cancer had developed. Currently, the search for novel anticancer drugs is being conducted among terrestrial plants, as well as in marine environments [1]. Plants have been used for centuries to treat diseases. In various parts of the world, several plants are consumed for their health benefits as a part of traditional folk medicine. The increase in the incidence of various types of cancer creates a need for new anticancer drugs. For example, in 2017, 1,688,780 new cancer cases and 600,920 cancer deaths are projected to occur in the United States [2]. Numerous anticancer drugs isolated from plant materials are tested on cells (including various cancer cell lines) and experimental animals after purification and then sent to clinical trials. In recent years, there has been a dynamic increase in the number of newly discovered natural compounds. In 2006, about 50,000 such substances were known, whereas, in 2014, the number of the newly discovered molecules increased to approximately 326,000. Among these, there were approximately 170,000 compounds in the toxicity class. In addition, there are 195,000 pharmacologically active compounds for which the interactions are quantitatively known [3]. Plants that have been used in traditional medicine for centuries have found application as sources of materials that possess high biological activity [4]. One approach is to obtain these substances through extractions from the plant materials. Another approach is to use biotechnological tools to produce plant-derived anticancer compounds. The substances of natural origin (e.g., from plants and aquatic animals) that exhibit antitumour properties belong to various groups of compounds, such as alkaloids, diterpenes, diterpenoquinone, purine-based compounds, lactonic sesquiterpene, peptides, cyclic depsipeptide, proteins, macrocyclic polyethers, etc. Sometimes, the cost of extraction of these substances from natural materials is much lower than the cost of their chemical synthesis. In the case of artemisinin, the low cost of the extraction process has been described: 250 kg of leaves yields 4 to 5 kg of raw artemisinin extract, which yields about 1 kg of pure artemisinine [5]. However, obtaining 50 grams of crude vincristine sulphate requires about 1 tonne of *Catharanthus roseus* leaves. However, vinblastine, which is present in *Catharanthus roseus*, occurs at a level that is 1000 times higher than vincristine and its cost is one-third of that of vincristine. Vinblastine is used as the parent drug to obtain vincristine [6]. In turn, paclitaxel is isolated from the yew tree or semisynthesized from its precursors, such as baccatin III and 10-deacetylbaccatin III, which are all isolated from this natural plant [7]. However, obtaining it from the tree bark leads to a reduction in the natural resources of these trees. Additionally, isolation from the Pacific yew tree yields a low concentration of the drug—only 100 mg of Taxol per kg of the bark [8]. Taxanes can also be extracted from the renewable and more readily available leaves of the European yew tree. To protect Taxus, methods for the synthesis of these compounds have been developed. Two independent research teams, Holton (1994) [9] and Nikolau (1994) [10], developed independent methods of paclitaxel synthesis [10,11]. In the year 1996, they were joined by Danishefsky (1996) [12], who developed another way to synthesize this taxane. Paclitaxel and docetaxel are used widely as monotherapies of breast cancer as well as in combination with other anticancer drugs, such as the combination of docetaxel with either cisplatin or carboplatin (also in ovarian cancer), and lately, bevacizumab [13,14,15]. Numerous natural products presented in this paper are modified by introduction of the nitroxide residue, which, in many cases, increases their anticancer activity [16]. The work describes the compounds of plant origin, their occurrence and variety of mechanisms of action, e.g., binding to microtubules, topoisomerase inhibition, binding to DNA, cell-cycle arrest, and apoptosis. Their properties have been tested on various cancer cell lines, experimental animals, and in human chemotherapy. The presented substances can be used alone or in combination with other antineoplastic agents of natural origin or with synthesized drugs. The work presents known anticancer drugs that have been approved for use, as well as new analogies and other less known substances with anti-cancer properties that may be considered as potential chemotherapy drugs. The uses, mechanisms of action, and doses of natural compounds from plants and the marine environment are presented in Table 1 and Table 2, respectively.

## 2. The *Catharanthus* Alkaloids

**Figure 1:**
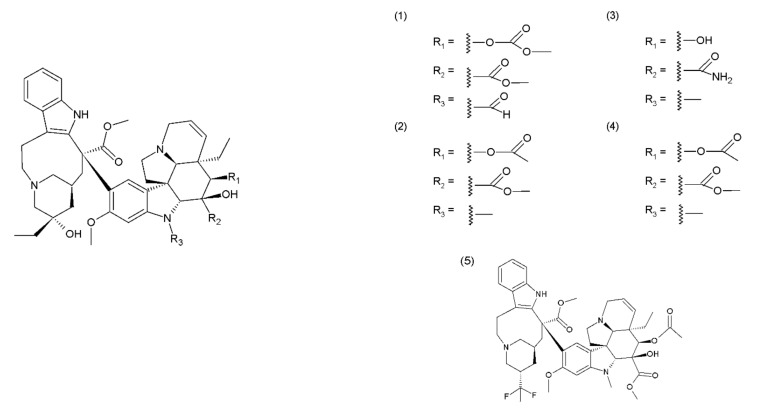
*Catharanthus* alkaloids: (**1**) vincristine, (**2**) vinblastine, (**3**) vindesine, (**4**) vinorelbine, and (**5**) vinflunine.

The *Catharanthus* (or Vinca) alkaloids (CAs or VAs) comprise a group of about 130 terpenoid indole alkaloids [5]. Vinblastine was the first alkaloid isolated by the Canadian scientists Robert Noble and Charles Beer from the Madagascar periwinkle plant in the 1950s. One group of medicines of plant origin is vincristine and its derivatives, which are heterodimeric (indoloid) alkaloids formed during the biosynthesis of vindoline and catharanthine that are present in pink *Catharanthus roseus*. Other *Catharanthus* species such as *C. longifolius*, *C. trichophyllus* and *C. lanceus* contain vindoline type alkaloids. These medicines demonstrate a cytostatic effect, destroying the living cells [17]. This group includes vincristine (1), vinblastine (2), anhydrovinblastine, and the semisynthetic derivatives vindesine (3), vinorelbine (navelbine) (4), and vinflunine (5) (the fluorinated analogue of vinorelbine) (Figure 1). The mechanism of the cytotoxic action of the *Catharanthus* alkaloids is associated with their effects on the microtubules. Microtubules are the cytoskeletal structures that are composed of the heterodimers of α-tubulin and β-tubulin, which exhibit dynamics that are manifested inter alia by the alternating elongation and shortening of their ends [18,19]. Microtubules form the mitotic spindle, which performs an important role in the process of mitosis. Therefore, microtubules are a suitable target for several anticancer drugs, including the vinca alkaloids, which bind to the β-subunit of the tubulin heterodimers [20,21]. The *Catharanthus* alkaloids demonstrate different mechanisms of action depending on their concentration. Low concentrations (<1 μmol) inhibit the microtubule dynamics and stabilise them, whereas, high concentrations (>1–2 μmol) disintegrate the microtubules and damage the mitotic spindle, leading to the inhibition of mitosis and consequently, to apoptosis [22]. *Catharanthus* alkaloids are among the most widely used chemotherapy reagents for various tumour therapies; however, they exhibit side effects. To reduce their toxicity and enhance the therapeutic efficiency of these compounds, liposome-entrapped drugs, chemical- or peptide-modified drugs, polymeric packaging drugs, and chemotherapy drug combinations were used [23]. *Catharanthus* alkaloids are used in chemotherapy to treat breast cancer, ovarian cancer, non-small cell lung cancer, and soft tissue sarcoma (orphan). They are also used in the treatment of acute lymphocytic leukaemia, malignant lymphomas, including Hodgkin’s lymphoma and non-Hodgkin’s lymphoma, multiple myeloma, idiopathic thrombocytopenic purpura and solid tumours, including metastatic testicular cancer, Ewing’s sarcoma, foetal rhabdomyosarcoma, primary neuroectodermal tumours (such as enucleus, nucleus, or neuroblastoma), Wilms’ tumour, and retinoblastoma [20,21]. Vinblastine and vinflunine are semisynthetic *Catharanthus* alkaloids [24]. For example, vinflunine is used in the treatment of bladder cancer. Vindesine is used mainly in the treatment of acute lymphocytic leukaemia and less frequently in the treatment of breast cancer, colorectal cancer, non-small cell lung cancer (NSCLC), and renal cancer. Vinorelbine is used in the treatment of non-small cell lung cancer. *Catharanthus* alkaloids are used in combination with other antineoplastic agents, for example, vinblastine is used with doxorubicin, bleomycin, and dacarbazine (standard chemotherapy for Hodgkin lymphoma) [25] and vincristine is used in combination with bleomycin, etoposide, doxorubicin, cyclophosphamide, procarbazine, and prednisone (Scheme of German Hodgkin Study Group developed a dose-escalated and accelerated combined modality regimen) [22], vinorelbine, and cisplatin (stage III A and stage III B non-small cell lung cancer) [26,27]. Lately, it has been shown that vincristine in combination with procarbazine and lomustine plus radiotherapy is more effective for glioma treatment than radiotherapy alone [28]. However, in addition to their valuable anticancer actions, these drugs, unfortunately, cause several side effects.

## 3. Viscum Album Extract

Another group of plant medicines includes viscotoxins (VT) and lectins isolated from the mistletoe plant (*Viscum album*); these medicines exhibit cytotoxic effects. Viscotoxins are present in the extracts isolated from the common mistletoe plant. Different forms of this drug may be obtained depending on the species of the tree on which the mistletoe grows. The medicinal preparations are available from a variety of host trees, such as oak, apple, pine, fir, willow, birch, lime, etc. The medicinal preparations obtained from *Viscum album* that are used in therapy are usually a mixture of the extracts obtained from various host trees. For example, one extract can come from various trees such as fir, apple tree, and pine, while another one can contain extracts from the apple tree, oak, and elm. Other medicinal preparations from mistletoe are used in the treatment of a different kind of cancer. The healing effects observed with the use of these extracts are usually more visible with the use of whole extracts rather than the use of purified mistletoe lectins and viscotoxins alone. *Viscum album* extract (VAE) contains the proteins known as lectins (mistletoe lectins, ML), namely ML–1, ML–2, and ML–3, the glycoprotein that binds to d-galactose and N-acetyl-d-galactosamine, viscotoxins, oligosaccharides, polysaccharides, and alkaloids [29]. VAE also contains flavonoids, such as quercetin and quercetin methyl ethers, kaempferol and a few of its methyl derivatives, and rarely, naringenin [30]. Viscotoxins are peptides with a molecular weight of approximately 5000 g/mol; they are rich in cysteine and belong to the class of plant thionins. They are toxic to a variety of cell types. It was demonstrated that viscotoxin and mistletoe lectin I exhibit toxic effects on three tumour cell lines–the Yoshida sarcoma cell line, the T-cell leukaemia cell line Molt4, and the myeloid leukaemia cell line K562. Yoshida sarcoma cells are more sensitive to viscotoxin while the human leukaemia cell line K562 is more sensitive to the mistletoe lectin [31]. In addition to the isolated lectins, viscotoxin oligosaccharides are also suspected to play a role in the biological activity of crude *Viscum album* extracts. VAE and its constituent compounds have strong cytotoxic effects on cancer cells in vitro. It has been reported that lectins are able to induce apoptosis. This process occurs together with the direct or indirect killing of the cells via damage to their cell membranes with the subsequent influx of calcium. It was also suggested that the damage caused to the membrane integrity may lead to the release of calcium from the cells and the activation of DNA-cleaving endonucleases [29]. It has been observed that the four bladder carcinoma cell lines (T–24, TCC-SUP, UM-UC–3, and J–82) are more sensitive to mistletoe preparations compared to the four breast carcinoma cell lines (MCF–7, MFM–223, KPL–1 and HCC–1937). In Dukes’ type C colorectal adenocarcinoma cell line Colo 320 HSR, VAE caused the activation of intrinsic (activated Caspase–2 and Caspase–9) and extrinsic (activated Caspase–2, Caspase–3 and Caspase–8) pathways of apoptosis [32]. In the colon cancer cell lines Caco–2 and HT–29, apoptosis was induced by the activation of only the mitochondrial pathway [33]. Similar results were obtained for the lung cancer cell lines MR 65, NCI-H125 and NCI-H82, where *Viscum album* extracts caused early dose-dependent cell cycle inhibition followed by apoptosis. However, the cytotoxicity test results revealed different responses in various breast cancer cell lines (Kpl–1, MCF–7 and Mfm–223) to the mistletoe preparations from different host trees [34]. Apoptosis was induced by the activation of its mitochondrial pathway [33]. The application of *Viscum album* extracts was demonstrated to increase the survival rate and tumour remission in various breast cancer and gynaecological cancer models especially in mice, while the application in rats demonstrated mixed results [35]. In vivo studies have demonstrated that the injection of *Viscum album* extract in patients with breast cancer leads to an increase in the number of neutrophils and the activation of the phagocytic cells. During a long-term therapy, another *Viscum album* preparation led to an increase in the number of B CD19+, Th CD4+, T CD8+, Tc CD8+ and CD28+, and NK CD16+/CD56+ cells within two to three months of therapy [36]. These drugs or extracts have found applications in the treatment of oral cavity, larynx, throat, and breast cancers. Moreover, the extracts were used in the adjuvant therapy for cancer because of their immunostimulatory and cytotoxic properties [37]. These effects are usually more evident with the use of the whole extracts, rather than the use of the purified mistletoe lectins and viscotoxins alone [29]. 

## 4. Taxanes

**Figure 2:**
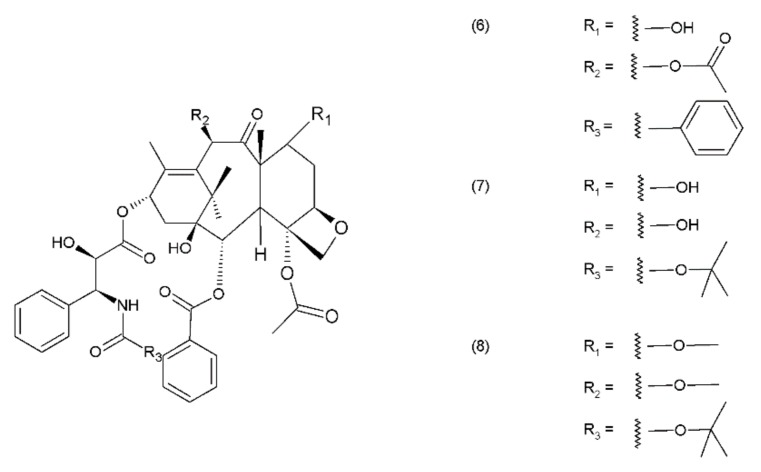
Taxanes: (**6**) paclitaxel, (**7**) docetaxel, (**8**) cabazitaxel.

Paclitaxel (PTX) (6) belongs to the group of drugs obtained from the European yew (*Taxus baccata*) and/or the Pacific yew (*Taxus brevifolia*) tree needles. It belongs to a group of compounds known as taxanes, which are mitosis inhibitors. Paclitaxel and its semisynthetic derivatives docetaxel (DTX) (7) and cabazitaxel (CTX) (8) are derived from 10-baccatin III or 10-deacetylbaccatin III (both contain characteristics of the taxane skeleton—three condenset homocyclic rings and one heterocyclic ring) (Figure 2). 

Although the full synthesis of paclitaxel has been described, there are still other methods for isolating taxanes from natural materials such as *Arabidopsis*, *Nicotian sylvestric*, or endophytic fungi, but these materials contain low concentrations of taxanes [38]. An interesting way to produce paclitaxel is plant cell fermentation. It is an effective and efficient method developed by Lin and colleagues that does not require the collection of natural products. The natural production of paclitaxel from Taxus is environmentally unsustainable and economically unfeasible. Lately, a method of synthesizing the precursor of paclitaxel 10-deacetylbaccatin III has been developed in the bioengineering sector [39]. Paclitaxel and docetaxel are used widely as monotherapies, as well as in combination with other anticancer drugs that inhibit mitosis and participate in cell apoptosis. However, both taxanes demonstrate differences in their toxicity profiles [40]. The anticancer activity of taxanes is similar to the action of vinca alkaloids and is associated with their effect on the microtubules, which are composed of heterodimers of α-tubulin and β-tubulin. Taxanes, however, constitute the second group of microtubule-interacting agents—microtubule-stabilising agents that stimulate the microtubule polymerization [18]. The processes of microtubule polymerisation and depolymerisation are essential for mitotic cell division. Taxanes, as opposed to vinca alkaloids, bind with high affinity to the inside of the microtubules. High concentrations of taxanes lead to microtubule polymerisation, while the *Catharanthus* alkaloids inhibit it. Taxanes are one of the most effective drug types used in the treatment of breast and ovarian cancers; they are also used to treat squamous cell carcinoma of head and neck [41]. Taxanes may be used as single agents or in combination with the anthracyclines, antimetabolites, and vinca alkaloids or even in combination with each other to provide benefits in the treatment of women with HR-negative metastatic breast cancer (MBC). To treat women with HER2-positive disease, the taxanes are administered along with trastuzumab, vinorelbine, or capecitabine. Trastuzumab is a monoclonal antibody that is also used in the treatment of breast cancer and metastatic gastric or gastroesophageal cancer. However, different levels of synergism have been observed between each taxane and trastuzumab [42]. The novel drugs that are being used in the metastatic therapy are the nanoparticle albumin-bound paclitaxel in MBC, and the propane-1,2,3-triol complex, which was initially approved for the treatment of relapsed ovarian cancer. The use of docetaxel in MBC therapy was ground-breaking, especially after the failure of using anthracyclines for this purpose. The application of docetaxel also yields better results than the use of mitomycin along with vinblastine. Given the high mortality rate of the disease, the treatment of breast cancer holds great importance in the field of medicine. The choice of treatment depends on the stage of cancer invasiveness (I, II, or III) from the non-invasive cancer stage (ductal carcinoma in situ). Metastatic breast cancer (stage IV) spreads from the nearby lymph nodes to other parts of the body. Paclitaxel is used in the treatment of breast and ovarian cancers, non-small cell lung cancer, and Kaposi’s sarcoma [43,44]. A combination of bevacizumab and paclitaxel exhibits synergetic effects, anti-tumour efficacy, and a satisfactory toxicity profile in tpatients with breast cancer and in patients with non-small cell lung cancer [45]. Paclitaxel may be used alone or in combination with other anticancer drugs, such as cisplatin or carboplatin [46,47]. In the treatment of ovarian cancer, dual therapy with docetaxel and camptothecin, as well as docetaxel in combination with gemcitabine, has demonstrated promising results as a second-line therapy. Similar results were obtained for triple-therapy treatment with docetaxel, carboplatin, and anthracycline–epirubicin [48]. The combination of a taxane, such as paclitaxel or docetaxel, with cisplatin or carboplatin is a standard therapy for ovarian cancer. However, in this dual therapy, carboplatin is used more often, as it has relatively fewer side effects with the same level of effectiveness. The application of cabazitaxel and its efficacy and safety when used in combination with prednisone in the treatment of metastatic castration-resistant prostate cancer (mCRPC) was demonstrated by Tsao et al. (2014) [49]. Cabazitaxel exhibited a significantly lower IC_50_ value than docetaxel. The authors suggested that a combination of taxanes with pigment epithelium-derived factor (PEDF) could serve as a novel strategy for castration-resistant prostate cancer (CRPC) chemotherapy [50]. Taxanes are effective drugs that are used to treat many cancers. However, like other anticancer drugs, they have side effects. The main problem is the development of cardiotoxicity, especially when they are given together with doxorubicin. However, this applies not only to the cardiac muscle, but also to other organs, like the kidneys, liver, and brain. It was shown that docetaxel induces oxidative stress in blood plasma in rats bearing mammary tumours [51,52]. Additionally, paclitaxel and docetaxel also generate oxidative stress in rat livers and brains [53,54]. 

## 5. Camptothecin

**Figure 3:**
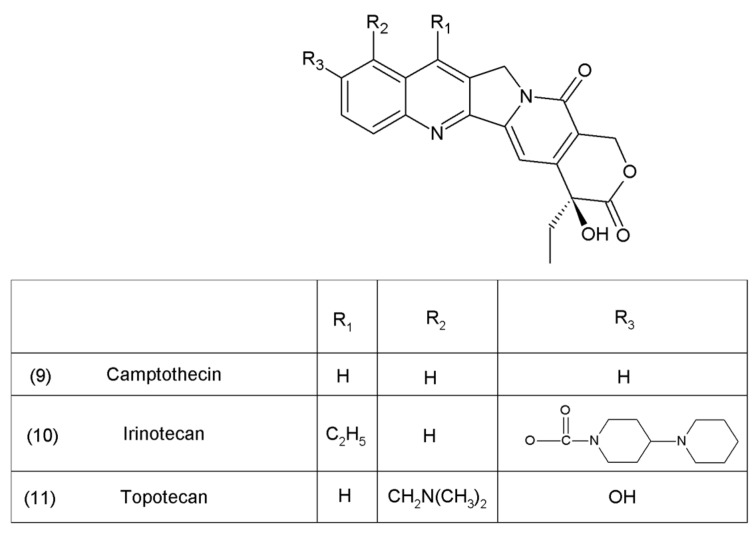
Camptothecin and its derivatives: (**9**) camptothecin, (**10**) irinotecan, (**11**) topotecan.

**Figure 4:**
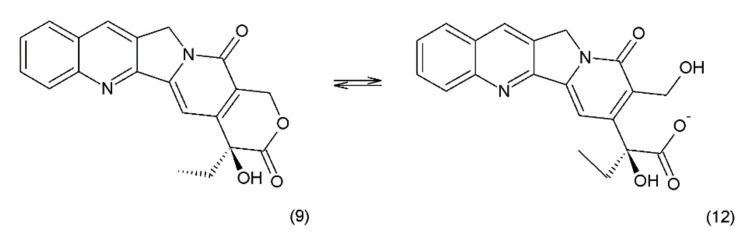
(**12**) Hydrolysis of the lactone ring in camptothecin.

Camptothecin (9) and its derivatives (Figure 3) belong to the pentacyclic group of quinoline alkaloids; these are isolated from *Camptotheca acuminata* and possess anticancer properties [17]. Camptothecin is present in the cortex, wood, and fruit of the *Camptotheca acuminata* plant. Camptothecin and its derivatives have also been obtained using different methods of synthesis [55,56,57,58,59]. The lactone rings of camptothecin are highly sensitive to hydrolysis which leads to the production of carboxylic acid derivatives (12) (Figure 4). Hydrolysis leads to a loss of the antitumour activity of camptothecin [60]. 

**Figure 5:**
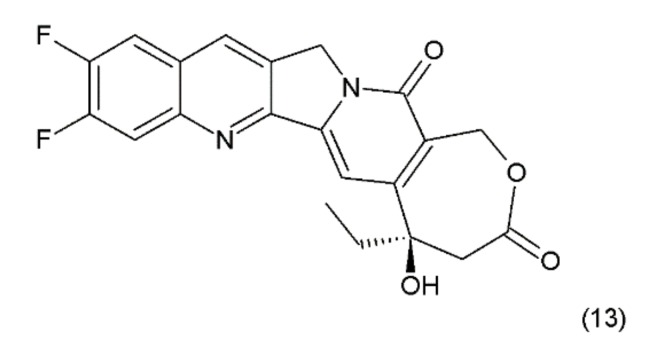
Stabilizing of the E ring by addition of methylene group; (**13**) diflomotecan.

Another disadvantage of this compound, as in the case of taxanes, is its poor solubility in water; however, its water-soluble semisynthetic derivatives, irinotecan (10) and topotecan (11), have found clinical application. The problem of hydrolysis in the camptothecin derivatives was solved by introducing a methylene group into their α-hydroxylactone ring, forming a stable, seven-membered ring in the structure (e.g., in diflomotecan) (13) which is not sensitive to hydrolysis (Figure 5). 

**Figure 6:**
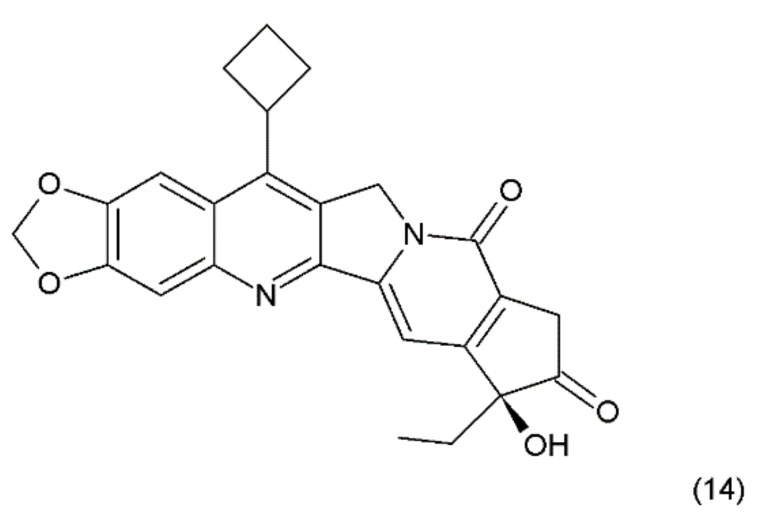
Stabilizing of the E ring by withdrawing of the lactonic group; (**14**) derivative of campothecin with the methylenodioxy ring and the substituent cyclobutane at position 7.

Another way to stabilise the α-hydroxylactone E-ring is to replace the lactone ring with a five-membered keto analogue of the ring. Such derivatives also retain high activity against topoisomerase I (TOP1) [61]. The derivative of camptothecin with a methylenedioxy ring and the substituent cyclobutane at position 7 demonstrates greater toxicity in different cancer cell lines than camptothecin (14) (Figure 6) [61,62]. 

**Figure 7:**
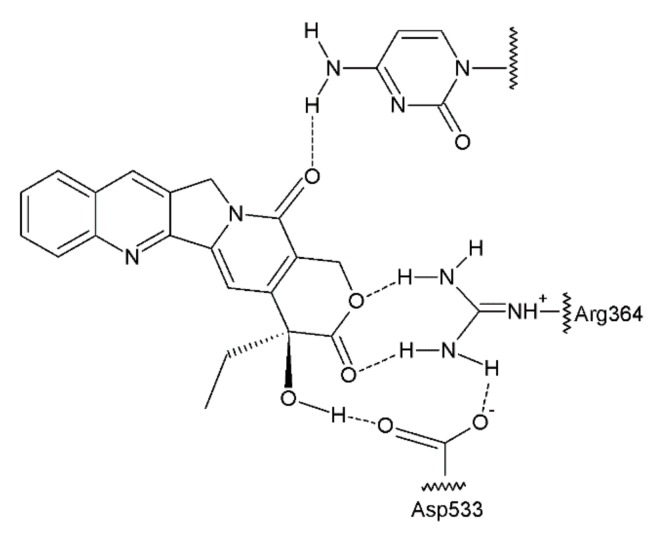
Camptothecin complex with topoisomerase I and DNA base [41].

The antitumour activity of this group of drugs is a result of the formation of reversible DNA strand breaks in the normal cell cycle. Camptothecin binds to a complex consisting of DNA and topoisomerase I, thus preventing the reassembly of the DNA strands of a single chain [63,64] (Figure 7). At that time, camptothecin intercalates between the nitrogenous bases in the DNA strands. 

**Figure 8:**
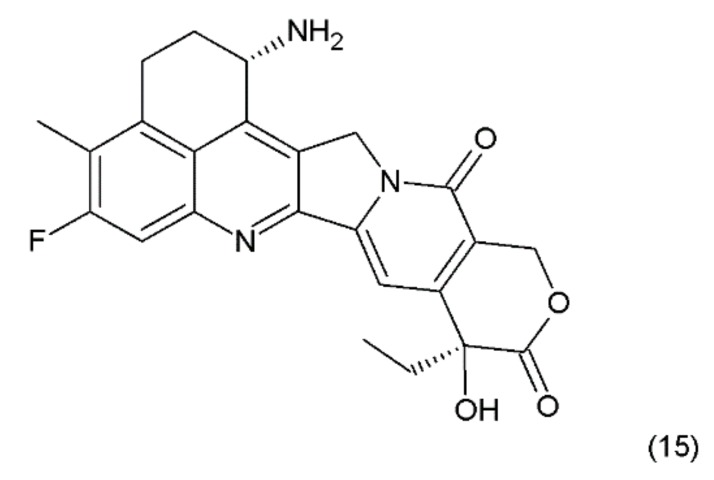
(**15**) Exatecan.

The combining of the camptothecin drug with the DNA–topoisomerase complex prevents the restoration of the bonds at the sites of nicks and damages the structure of the double DNA chains. The double-strand breaks introduced by the replication process are probably the main reason for the DNA damage in the tumour cells that are treated with camptothecin and its derivatives, which are the drugs that specifically act on the S phase of the cell cycle. Another derivative of camptothecin is the hexacyclic exatecan (15), which has a 6-membered ring at positions 7 and 9, a methyl group at position 10, and a fluorine atom at position 11 (Figure 8). 

It was reported that the water-soluble exatecan is more effective as an anticancer drug than topotecan [65]. Currently, several derivatives of camptothecin are known, and only a few of them are discussed in this paper. Recently, Liu et al. (2015) [66] presented 187 novel camptothecin derivatives, several of which are characterised by high toxicity in relation to human tumour cell lines, such as lung carcinoma (A–549), prostate carcinoma (DU–145), nasopharyngea (KB), and vincristine-resistant nasopharyngea (KB-Vin). A camptothecin derivative, topotecan, is used in the first-line and second-line treatments as a chemotherapeutic agent for metastatic and small-cell lung cancers [67,68]. In the European Union and the USA, it was accepted as the only drug in the second-line chemotherapy for recurrent small-cell lung cancer (SCLC). Irinotecan is used in the chemotherapy of gastrointestinal cancers [69,70,71]. This drug, in combination with 5-fluorouracil and leucovorin, is used as a chemotherapy for metastatic colorectal cancer [72]. In the first-line and second-line treatments of this cancer, combinations of bevacizumab and irinotecan have also been used [73]. Both of those camptothecin derivatives are used in the treatment of cervical cancer, gliomas, and sarcomas such as Ewing’s sarcoma and rhabdomyosarcoma [74,75]. In addition, both of those camptothecin derivatives have been used in clinical trials in the following cases: small-cell and non-small cell lung, breast, and hepatocellular carcinomas; mesotheliomas of the ovaries, cervix, oesophagus, stomach, pancreas, and kidney; and malignant neoplasms of the head and neck. Irinotecan and cisplatin have been used in the treatment of SCLC or extensive-stage SCLC [76]. Irinotecan is also used in the first-line and second-line treatments in combination with gemcitabine, cisplatin, and docetaxel in patients with metastatic pancreatic cancer [77,78]. Higher toxicity was observed when exatecan was used with gemcitabine, compared to the use of gemcitabine alone. The combination of these two drugs was, however, not superior to the use of gemcitabine alone in the first-line treatment of advanced pancreatic cancer, in terms of the overall survival [79]. Certain other researchers have used irinotecan in combination with gemcitabine and celecoxib in the treatment of patients with advanced pancreatic cancer [80]. Gemcitabine has been used, either alone or in combination with irinotecan, leucovorin, and 5-fluorouracil, in the first-line treatment of the patients with metastatic pancreatic adenocarcinoma [81].

## 6. Combretastatin

**Figure 9:**
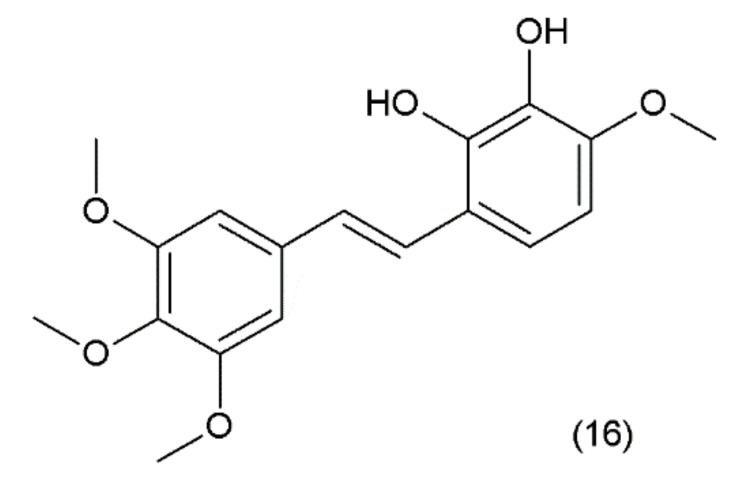
(**16**) Combretastatin A-1.

Combretastatin (16) is a stilbene derivative that is present in the *Combretum caffrum* tree, also known as the “African willow”, which grows in the Southeastern region of Africa. As a stilbene derivative, it may exist in trans (e.g., combretastatin A–1 (CA–1)) and cis (e.g., combretastatin A–4 (CA–4)) forms (Figure 9). 

Combretastatin A-4 and its derivatives were synthesized by Pettit and colleagues in 1987 [82]. Recently, the synthesis of new derivatives of podophylotoxins was performed and these were tested on skin carcinoma A431, human cervix adenocarcinoma HeLa, breast cancer cell lines: MCF7, MDA-MB-231, lung carcinoma A549, and ovarian cancer cells SKOV. These compounds efficiently inhibit tubulin polymerization with IC_50_ values below 1 μM [83]. Combretastatins are microtubule-targeting drugs similar to taxanes and vinca alkaloids. These compounds belong to the class of vascular disrupting agents (VDAs), which inhibit the angiogenesis of the tumour blood vessels. The mechanism of action of combretastatin as an antineoplastic compound is associated with its binding to β-tubulin at what is known as the colchicine site, causing the destabilisation of the microtubules. Inhibition of the tubulin polymerisation prevents the cancer cells from producing microtubules, which leads to the inhibition of cell proliferation, resulting in the process of apoptosis and/or the cytotoxic mechanism of action. While the combretastatin A–4 isomer is more potent as a tubulin binder, the combretastatin A–1 trans isomer exhibits a stronger cytotoxic effect. The molecules that have an amino group at position 2 either in ring A or ring B and a hydroxyl group at the 3’ position are highly active [84]. On the other hand, the presence of a hydroxyl or methoxy group at the 4’ position also provides cytotoxic ability. It has been demonstrated that combretastatin A4 inhibits proliferation, migration, and invasion, and promotes apoptosis in the human thyroid papillary carcinoma cell line (TPC1) [85]. A single intraperitoneal (i.p.) dose of combretastatin A4 resulted in 30% volume destruction (average) of the metastatic mass in liver metastasis within 24 h of the administration of the drug [86]. Combretastatins are also used in combination with other anticancer compounds in the treatment of anaplastic thyroid cancer (ATC). Combretastatin A–4 phosphate (CA4P) has been used in a series of combinations, first with paclitaxel and manumycin A (a selective farnesyltransferase inhibitor) and the second with paclitaxel and carboplatin. Both drug combinations were used in the treatment of ATC, which is extremely aggressive and has no effective available treatment to date. It was demonstrated that both triple-drug combinations exhibited excellent antineoplastic activity against anaplastic thyroid cancer [87]. The addition of CA4P to the combination therapy with paclitaxel and carboplatin was well-tolerated and led to a higher response rate in this patient population in comparison to chemotherapy without the addition of CA4P [88].

## 7. Podophyllotoxin

**Figure 10:**
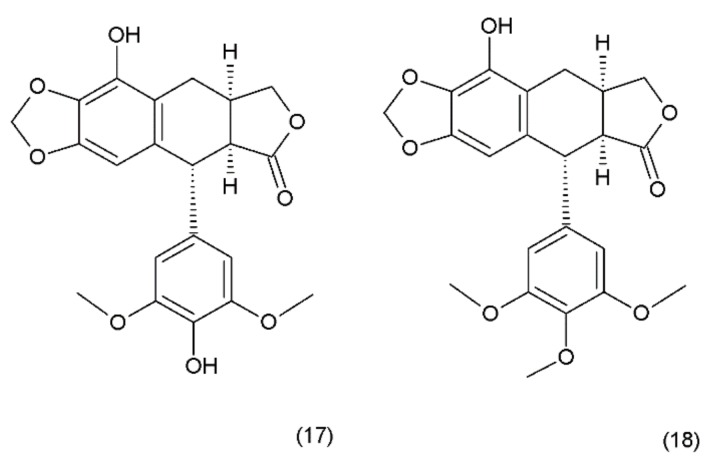
Pelatins: (**17**) α-peltatin, (**18**) β-peltatin.

Podophyllotoxin (PPT) (19) is a toxin lignan isolated from the Berberidaceae family (i.e., *Podophyllum*). The resin known as podophyllin was obtained from the *Podophyllum peltatum* species found in North America. PPT was extracted from *Podophyllum emodi* resin from Asia [89]. Podophyllotoxin also occurs in the plants of the *Linum* amd *Juniperus* species and in *Podophyllum versipelle* [90]. *Podophyllum peltatum* plants also contain α-peltatin (17), β-peltatin (18) (Figure 10), and their corresponding glycosides, along with podophyllotoxin-α-d-glucoside. The first synthesis of podophyllotoxin was performed by Gensler and Gatsonis in 1962. 

**Figure 11:**
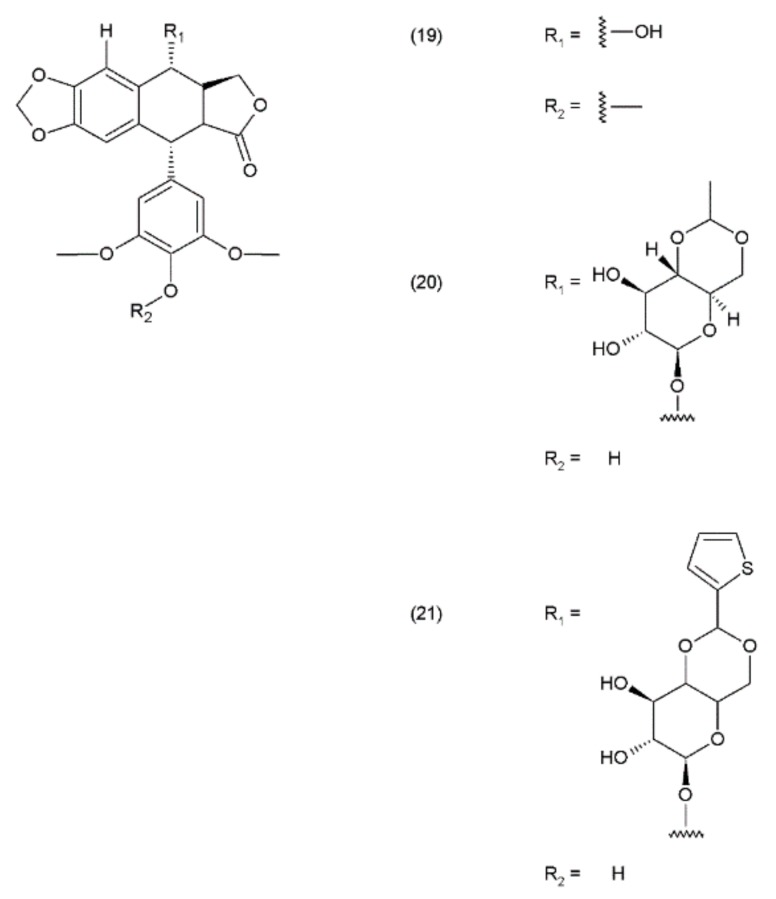
Podophyllotoxin derivatives: (**19**) podophyllotoxin, (**20**) etoposide, (**21**) teniposide.

Podophyllotoxin and its derivatives are aryl tetralin lactone (condensed tetracyclic ring with an aryl substituent) compounds that contain the lactone ring in its trans conformation. PPT and its derivatives exhibit significant biological activity as antiviral agents and as antineoplastic drugs. Podophyllotoxin and its glycosides exhibit a very strong cytostatic effect, as they disrupt the organisation of the karyokinetic spindle. These compounds attach to the colchicine domain of tubulin and inhibit tubulin polymerisation. In addition, the podophyllotoxins cause single-strand and double-strand breaks in DNA through their interactions with DNA topoisomerase II, inducing cell-cycle arrest in the G2-phase of the cell cycle. This activity is mediated through the formation of a stable complex with DNA and topoisomerase II. Etoposide (20) and teniposide (21) are the semisynthetic derivatives of podophyllotoxin and exhibit cytostatic activity (Figure 11). 

In contrast to podophyllotoxin, etoposide and teniposide do not inhibit the tubulin polymerisation; instead, they belong to the group of phase-specific drugs (work in interphase) [91]. In primary brain tumour cases, including paediatric patients with refractory or relapsed brain tumours, as a salvage treatment, the majority of the patients receive etoposide as a part of a multi-agent regimen that includes cisplatin, carboplatin, cyclophosphamide, vincristine, and etoposide [92,93]. Etoposide and teniposide stabilise the intermediate covalent complexes formed between topoisomerase II and the cleaved DNA as well as the induction of DNA damage, which arrests the cell cycle in metaphase and leads to apoptotic processes [94,95]. Podophyllotoxin is also used for the semisynthesis of anticancer drugs, such as etoposide, tenioposide, azatoxin, and etopophos as well as NK611, GL331, TOP-53, and tafluposide [94,96]. PPT derivatives are used in the treatment of numerous cancers, such as lymphomas, neuroblastomas, sarcomas, testicular and ovarian cancers, brain tumours, gastrointestinal cancer, cervical carcinomas, osteosarcomas, nasopharyngeal carcinomas, colon cancer, breast cancer, prostate cancer, small-cell lung cancer, and testicular carcinomas [97]. Podophyllotoxin is a highly toxic compound, and therefore, it is rarely used in cancer therapy [94]. In contrast, its derivatives, such as etoposide and teniposide, are used in therapy for lung cancer, testicular cancer, lymphomas, and gliomas [91]. PPT derivatives are also used in the combination therapy, for example, in combination with cisplatin in the first-line chemotherapy for small-cell lung cancer or extensive-stage SCLC [75]. However, in certain studies that have been conducted on the effectiveness of chemotherapy in the treatment of small-cell lung cancer, a combination therapy with cisplatin, etoposide and irinotecan drugs was compared to the topotecan monotherapy. It was demonstrated that the combination chemotherapy could serve as the standard second-line chemotherapy in the sensitive recurrent SCLC [98]. Moreover, podophyllotoxin also exhibits anti-multidrug resistance (anti-MDR) potential against a variety of drug-resistant tumour cells. Recently, 766 novel derivatives of podophyllotoxin have been reported. Several of these have been tested on the cancer cell lines, where they exhibited high anticancer activity and are very much expected to be tested as anticancer drugs in the undergoing clinical trials [99]. Recently, Zhang et al. (2017) [97] demonstrated that 76 novel derivatives of podophyllotoxin exhibit biological activity, including the anticancer properties. Deoxypodophyllotoxin (DPT), which is closely related to podophyllotoxin structurally (lack of hydroxyl group in ring C), is a potent antitumour and anti-inflammatory agent. DPT is isolated from *Juniperus communis* (common Juniper) as aryltetralin lignan deoxypodophyllotoxin as potent inducers of apoptosis in malignant MB231 breast cancer cells [100].

## 8. Geniposide and their derivatives

**Figure 12:**
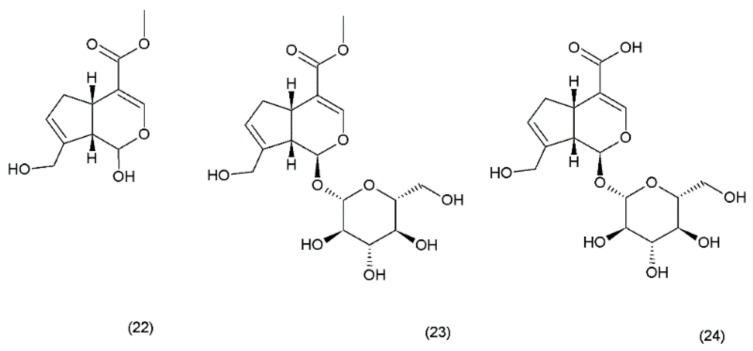
Genipin derivatives: (**22**) genipin, (**23**) geniposide, (**24**) geniposidic acid.

Geniposide (GS) occurs in nearly 40 species belonging to different families; however, the most famous sources of geniposide are the fruits of *Gardenia jasminoides* Ellis (Rubiaceae), which have been used in Chinese medicine for centuries. Numerous other species of this genus and the other members of the Rubiaceae family are known to contain the geniposide. Currently, about 90 different derivatives of the geniposide backbone are known. Genipin (22) is an aglycone that is formed by the hydrolysis of geniposide (23) which occurs in the same plant material along with the other derivatives, such as geniposidic acid (24) (Figure 12) [101]. Other biologically active iridoid derivatives was described by Liu and Lou in 2007 [102]. 

Other derivatives based on the iridoid backbone are most often the glycosides or esters of aromatic acids. It has been demonstrated that genipin and geniposide exhibit antidiabetic effect, as well as neuroprotective effects in neurodegenerative diseases (e.g., Alzheimer’s disease) [103,104]. The derivatives of iridoid glycoside geniposide are a relatively novel group of compounds with antitumour activity, which have been recently submitted to a critical review. The work discussed the influence of this group of compounds on the development of tumour cells and their mechanisms leading to the death of cancer cells. The work also discussed the pro-oxidant and antioxidant mechanisms of these compounds, the generation of reactive oxygen species, cell-cycle regulation, and the pro-inflammatory properties of these compounds [105,106]. Geniposide has been reported to inhibit the hydroperoxide and myeloperoxidase formation caused by 12-O-tetradecanaoylphorbol-13-acetate (TPA) in addition to inhibiting TPA-induced skin tumours in female CD–1 mice in vivo [107]. It has reported that genipin exhibits a strong apoptotic cell death effect in human non-small-cell lung cancer H1299 cells. Genipin caused increased levels of Bax in response to p38MAPK signaling, which led to the initiation of the mitochondrial death cascade [108]. Recently, the properties of geniposide and its derivatives as well as their toxicology and pharmacology have been described [101]. 

## 9. Colchicine

**Figure 13:**
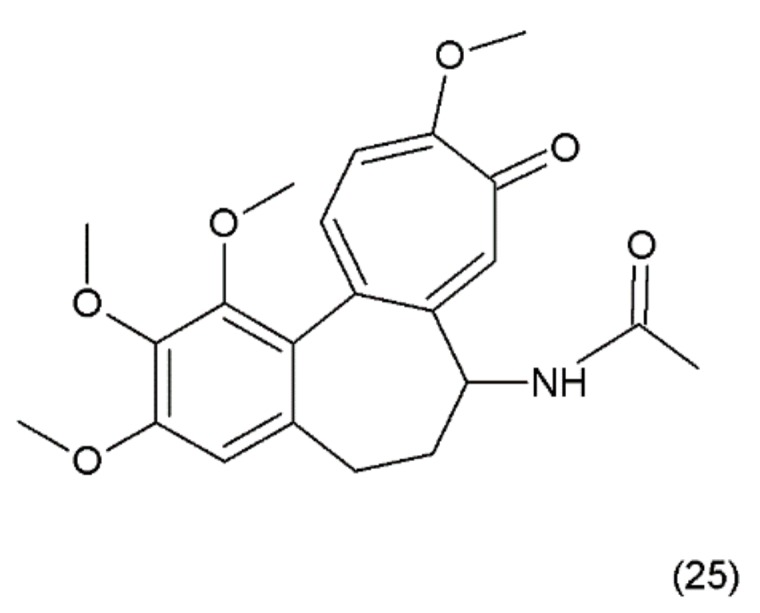
(**25**) Colchicine.

Colchicine (25) (Figure 13) is an alkaloid that is extracted from the plants of the *Colchicum* genus (e.g., the autumn crocus, *Colchicum autumnale*, also known as “meadow saffron”). Colchicine was synthesized for the first time by Van Tramalen and collaborators (1959) [109]. 

Similar to vinca alkaloids, the mechanism of action of colchicine is based on the depolymerisation of the microtubules at high concentrations and the stabilisation of the microtubule dynamics at low concentrations. Most of the antimitotic drugs, such as podophyllotoxin and combretastatin, bind to the colchicine domain of tubulin and interfere with the in vitro and in vivo assembly of the microtubules in the lowest-possible effective concentrations that stabilise the microtubule dynamics. Colchicine may modify the voltage-dependent anion channels of the mitochondrial membrane and increase the cellular free tubulin to limit mitochondrial metabolism in the cancer cells [110]. Colchicine is a highly toxic and very cheap alkaloid and has been used in medicine for a long time. For example, the antiproliferative effect of colchicine on hepatocellular carcinoma cells is approximately 200-fold less than that of epirubicin. It has been demonstrated that a low colchicine concentration is clinically acceptable in the palliative treatment of hepatocellular carcinoma [111] and cholangiocarcinoma [112]. The inhibition effect of 6 ng/mL colchicine on the growth of hepatocellular carcinoma cells was shown to be equivalent to the effect of 1 mg/mL epirubicin, which is the maximum plasma concentration of this drug used in patients. It has been demonstrated that colchicine exhibits inhibitory effects on the proliferation of two human gastric cancer cell lines (AGS and NCI-N87). Colchicine has also been demonstrated to inhibit tumour growth in nude mice [113]. In patients with gout who were treated with colchicine, the incidence of all-cause cancers was significantly lower in comparison to patients who did not receive the colchicine treatment [114]. Similar to the vinca alkaloids, colchicine also depolymerises the microtubules when it is used at high concentrations and stabilises the microtubule dynamics when used at low concentrations. Colchicine inhibits the microtubule polymerisation by binding to the microtubule ends, rather than binding to the soluble tubulin pool. However, the free colchicine itself probably does not bind directly to the microtubule ends. Instead, it first binds to the soluble tubulin, which induces gradual conformational changes in the tubulin and ultimately, forms a poorly reversible tubulin–colchicine complex, which then copolymerises into the microtubule ends in small numbers along with numerous free tubulin molecules [112].

## 10. Artesunate

**Figure 14:**
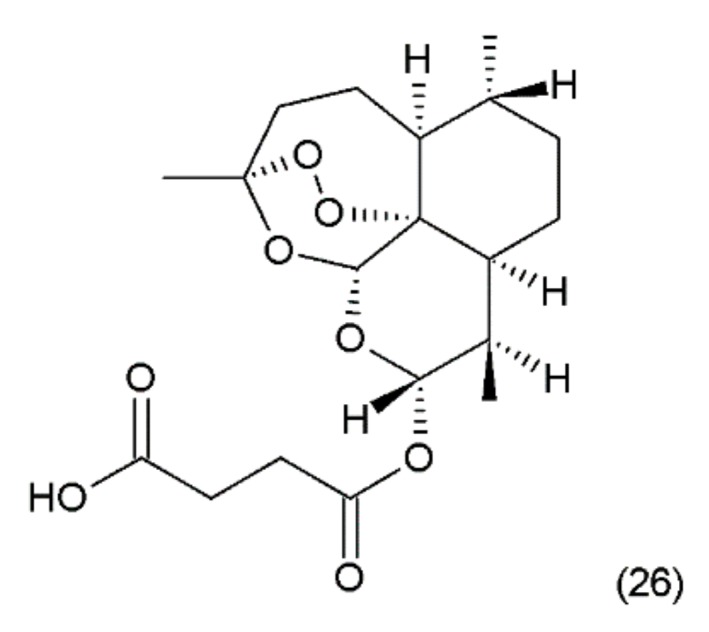
(**26**) Artesunate.

Artesunate (26) (Figure 14) is a semi-synthetic derivative of artemisinin isolated from a Chinese plant *Artemisia annua L.* medicinal (Asteraceae) [5]. Artemisinin was synthesized by Yadav and co-workers (2003) [115]. It was used as an antimalarial drug. It has been shown that artesunate exhibits activity against certain types of cancer through an anti-angiogenic mechanism. Artemisinin and its derivatives inhibit the growth and proliferation of cells and selectively kill tumour cells. Recently, many new artemisinin derivatives have been described, and it is possible that many of them will be effective against cancer [116].

It also demonstrated activity against chronic leukaemia in vitro and in vivo. It has been indicated that these compounds have low toxicity and are promising agents in antileukemia chemotherapy [117]. Additionally, artemisinin is not transported with P-glycoprotein, so it is not involved in multidrug resistance [118]. After the administration of artemisinin to rats for several weeks, severe adverse effects were not found [119]. Other studies compared the efficacy and toxicity of artesunate combined with vinorelbine and cisplatin, as well as artesunate alone, in the treatment of advanced non-small cell lung cancer (NSCLC). Artesunate combined with vinorelbine and cisplatin can elevate the short-term survival rate and prolong the mean survival time of patients, without extra side effects [120]. Zhao et al. (2013) [121] reported that artemisinin is cytotoxic to retinoblastoma cell lines with low cytotoxicity on normal retina cell lines. Artemisinin therapy has also been used in clinical trial studies without serious adverse effects [122]. Artemisinin was also combined with daunorubicin to produce overall antitumour efficacy without signs of toxicity in mice [123]. No adverse effects have been observed in the treatment of breast cancer either [124]. Artesunate exhibited antitumour activity against human pancreatic cancer through the induction of apoptosis. This effect depended on the stage of differentiation and was more effective against less diverse cells. Additionally, it enhanced the effects of gemcitabine in the control of pancreatic cancer [125]. Artesunate was reported to be effective against chemoresistant neuroblastoma cells. This drug was shown to induce apoptosis and reactive oxygen species in neuroblastoma cells [126].

## 11. Homoharrigtonine

**Figure 15:**
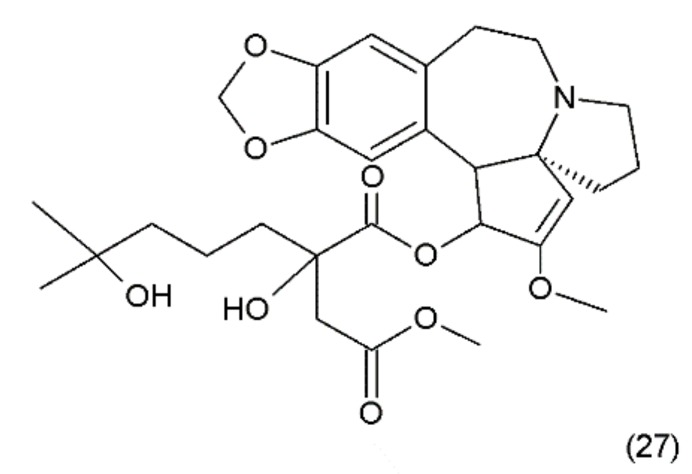
(**27**) Homoharrigtonine.

Another anticancer drug is homoharrigtonine (Omacetaxine mepesuccinate) (27) (Figure 15), which was first isolated in 1963 from several *Cephalotaxus* (Cephalotaxaceae), including *C. harringtonia* K. Koch, and *C. haianensis qinensis*, all of which are used in traditional Chinese medicine. The first synthesis of homorringtonine was performed by Hiranuma and Chudlicky (1982) [127]. These compounds were used in the treatment of various types of cancer, particularly in leukaemia and breast cancer. The action of these drugs is connected with the block of synthesis in the peptidyl transferase centre and leads to cell apoptosis [128]. The use of homoherringtonin in chronic myeloid leukaemia made it possible to achieve remission in 92% of patients, with a favourable cytogenetic response in 68%. Current research is aimed at increasing the therapeutic effect by combining homoharringtonine with IFN-α and cytarabine in low doses in patients with worse prognoses [129].

## 12. Salvicine

**Figure 16:**
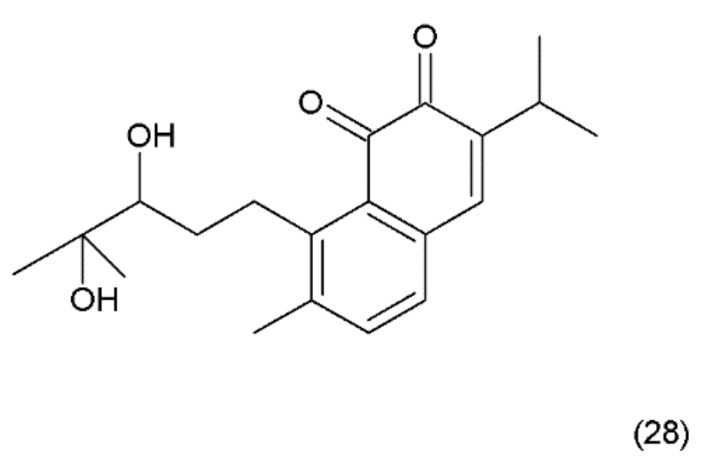
(**28**) Salvicine.

Salvicine (28) (Figure 16) is a modified diterpenoquinone derivative isolated from the Chinese herb *Salvia pronitis* Hance (Labiatae). Salvicine was chemical synthesized by Sheng et al. in 1999 and it has shond potent inhibitory activity against a wide spectrum of human tumour cells in vitro and in mice bearing human tumour xenografts [130]. Salvicine and its derivatives are nonintercalative topoisomerase II poisons that exhibit strong antitumour effects in vitro and in vivo and a broad spectrum of anti-multidrug resistant activity [131]. Salvicine initiates the break of two strands of DNA by facilitating TOP2 activity, inhibiting re-ligation, which is associated with the inhibition of tumour growth. In human cancer cells, salvicine induces damage to specific DNA genes, leading to apoptosis. Additionally, reactive oxygen species have been shown to play a key role in the salvicine-induced cellular response, including TOP2 inhibition, DNA damage, circumventing MDR and tumour cell adhesion inhibition [130]. It was reported that new series of salvicine derivatives demonstrated potent cytotoxicity against tumour cell lines [132].

## 13. Elipticine

**Figure 17:**
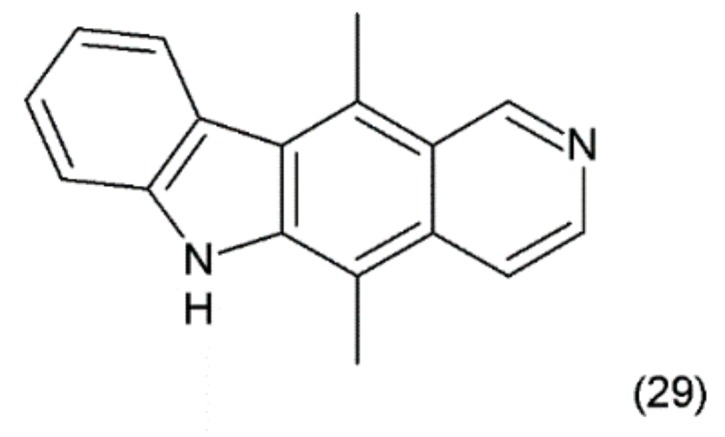
(**29**) Ellipticine.

The ellipticine (5,11-dimethyl-6H-pyrido-(4,3-b)-carbazole) (29) (Figure 17) is an alkaloid found in *Apocyanaceae* species. It was isolated from the leaves of *Ochrosia elliptica* Labill. Synthetic ellipticine was obtained by Woodward et al. (1959) [133], and together with its more soluble derivatives, it exhibits considerable antitumoural activity. 

Ellipticins bind strongly to DNA, forming covalent bonds, and/or to TOP2 [134] and lead to disruption of the cell cycle by regulating the expression of some kinases, such as cyclin B1 and Cdc2 [135]. In addition, they can phosphorylate the latter cyclin, inducing apoptosis and generation of cytotoxic free radicals [136]. Ellipticine is also an inhibitor or inducer of biotransformation enzymes. Through modulation, this metabolism indicates genotoxic and pharmacological effects. Ellipticine is cytotoxic in human breast cancer cells (MCF-7), leukemia (HL-60 and CCRF-CEM) cells, neuroblastoma (IMR-32, UKF-NB-3 and UKF-NB-4) cells, and glioblastoma cells (U87MG) [137]. Its powerful antitumour activity, e.g., pharmacological efficiency and/or genotoxic side effects, are dependent on its cytochrome P450 (CYP)- and/or peroxidase-mediated activation to species forming covalent DNA adducts [138]. In clinical trials, ellipticine has been observed to induce the remission of tumour growth.

## 14. Roscovitine

**Figure 18:**
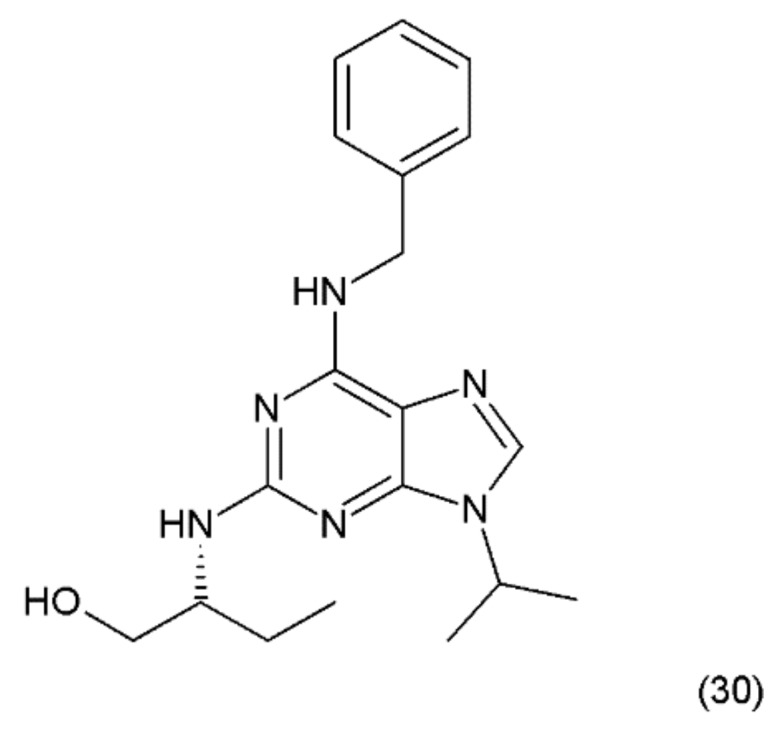
(**30**) Roscovitine.

The next anticancer compound is a purine-based, semisynthetic derivative of R-roscovitine (30) (Figure 18), which is derived from olomoucine and was isolated from the cotyledons of *Raphanus sativus L.* (Brassicaceae). 

Roscovitine was synthesized by Meijer et al. (1997) [139] and is one of the most promising members of the cyclin-dependent kinase (CDK)-inhibitor family. It has been shown that roscovitine, a potent CDK/cyclin E inhibitor, exhibits a wide spectrum of actions against cancer, neurodegenerative diseases, viral infections, and glomerulonephritis. Roscovitine was used in phase-2 clinical testing against lung and breast cancer, and phase-1 clinical trials against glomerulonephritis. Seliciclib has been shown to inhibit RNA-polymerase-II dependent transcription and down-regulation of the protein MCL1 [140]. Olomoucine is a cyclin-dependent kinase inhibitor (CDK4) [141]. In turn, roscovitine specifically inhibits CDK5 activity with concomitant inhibition of MDA-MB231 cell proliferation and induction of apoptosis. In contrast, olomoucine does not affect MDA-MB231 cell proliferation and apoptosis; however, roscovitine-mediated inhibition of proliferation is irreversible [142]. These data suggest that CDK5 may have a significant role in the regulation of breast cancer cell proliferation and apoptosis. However, roscovitine has a stronger effect on it than olomoucine [62]. The probable mechanism of action of roscovicine and its derivatives is related to the inhibition of cyclin-dependent kinase activity, which preferentially inhibits numerous target enzymes, such as CDK1, CDK2 and CDK5, leading to cell-cycle arrest in the G1 and G2 phases [142,143]. It was reported that roscovitine inhibits RNA synthesis in human cells, probably by mechanisms other than CDK inhibition [144]. A synergistic mechanism of action of roscovitine with the farnesyltransferase inhibitor during the induction of apoptosis in HL-60 cells was reported, since similar synergy was observed with a leukemic cell line (CEM) and a prostate cancer cell line (LNCaP) [144,145]. Wortmannin, a phosphatidylinositol-3-kinase (PI3K) inhibitor, was shown to enhance the effects of roscovitine by causing a reduction in the mitochondrial transmembrane potential, increasing cytochrome c release and activating caspase-3, as well as enhancing the activation of Bax and Bad [143]. These results suggest that roscovitine inhibits Ang II-induced angiotensinogen expression by hindering the activation of ERK1/2 and c-Jun [146]. Roscovitine was shown to exert clear dose-dependent anti-proliferative and pro-apoptotic effects in glioblastoma A172 [147]. It was reported that roscovitine has a synergistic effect with other anticancer drugs, such as doxorubicin, paclitaxel, 5-fluorouracil, vinblastine, alemtuzumab, trastuzumab, cisplatin, irinotecan, etoposide, and tamoxifen, as well as with radiation [148].

## 15. Maytansin

**Figure 19:**
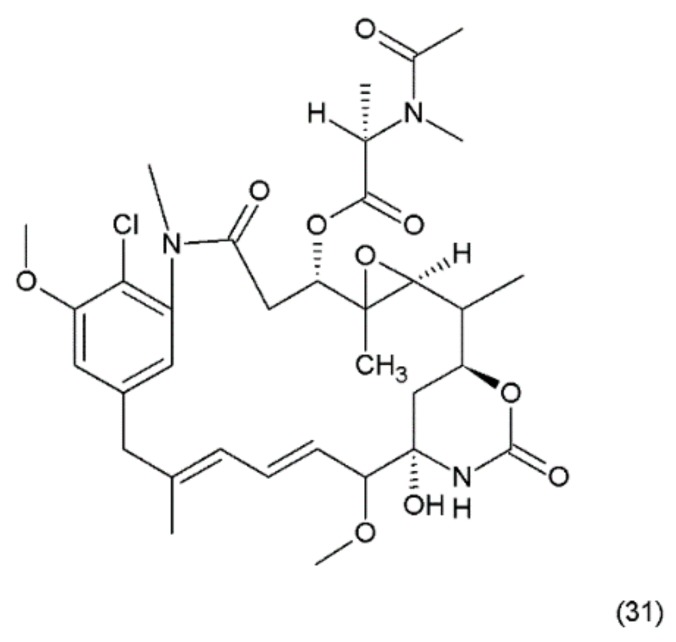
(**31**) Maytansin.

Maytansin or maytansine (31) (Figure 19) is a cytotoxic agent that was isolated in 1970s from the Ethiopian plant *Maytenus serrata* (Celastraceae) or *Maytenus ovatus* and it was synthesized by Corey and co-workers (1980) [149]. Maytansin and its derivatives belong to the natural product group of maytansinoids, which have antimitotic effects that are attributed to their ability to inhibit microtubule assembly by binding to tubulin near the vinblastine binding site [150].

The mechanism of action of maytanasin is the same mechanism as in the vinca alkaloids. Maytansin was used as an effective drug (in vivo) against Lewis lung carcinoma and B16 murine melanocarcinoma solid tumours and has antileukemic activity against P388 murine lymphocytic leukaemia. The maytansinoids belong to the ansamycin group of natural products containing ansamacrolide structures attached to a chlorinated benzene ring. Many maytanasin derivatives were synthesized by Cassidy et al. (2004) [151]. Although maytansin is cytotoxic to cancer cells, its clinical application has been hampered by severe side effects and poor efficacy [151]. However, its derivatives, especially maytansin analogues conjugated to antibodies (maytanosoids), are used in various types of cancer chemotherapy [152,153]. However, maytansinoids were shown to be 20 to 100 times more potent than vincristine in vitro [154]. The mechanism of action of maytansinoids is related to their high affinity to receptors expressed on the surfaces of cancer cells. The HuN901 monoclonal antibody conjugate with maytansin showed high affinity for CD56 [155]. This drug has been used with good efficiency in primary multiple myeloma (MM) cells showing the expression of CD56. Another maytansinoid conjugate, trastuzumab-DM1, yielded promising results in patients with metastatic breast cancer during a phase II clinical trial [153].

## 16. Thapsigargin

**Figure 20:**
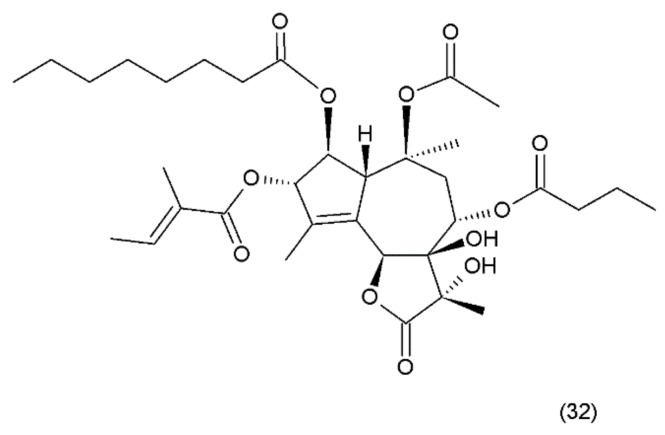
(**32**) Thapsigargin.

Thapsigargin (TG) (32) (Figure 20) is a lactonic sesquiterpene that was isolated from the roots of *Thapsia garganica L*. (Apiaceae) collected from Ibiza island [142]. Thapsigargin, which was synthesized by Ball et al. (2007) [156], and its derivative represent a group of antineoplastic drugs. Thapsigargin is an inhibitor of sarcoplasmic/endoplasmic reticulum calcium adenosine triphosphatase (SERCA) in the sarco/endoplasmic reticulum. Thapsigargin leads to an increase in the intracellular calcium concentration by blocking the ability of the cell to pump calcium into the sarcoplasmic and endoplasmic reticulums [157].

Thapsigargin induces perturbations in calcium homeostasis and an increase in nitric oxide concentration induces apoptosis and cell death [158]. A disturbance in calcium concentration enhances endoplasmic reticulum stress, which, in turn, leads to caspase activation, the release of apoptotic factors from the mitochondria, and direct activation of calcium-dependent endonucleases that cleave cellular DNA, resulting in cell death [159,160]. Thapsigargin specifically inhibits the fusion of autophagosomes with lysosomes, the last step in the autophagic process leading to cellular death [161]. Thapsigargin is useful in experiments examining the impacts of increasing cytosolic calcium concentrations. TG has been shown to induce apoptosis in quiescent and proliferating prostate cancer cells where standard anti-proliferative chemotherapy is ineffective, although it did not show selectivity for those cells. In clinical studies, it was shown to be associated with a carrier peptide to produce a prodrug activated by prostate-specific membrane antigen (PSMA). Mipsagargin is a novel derivative of thapsigargin-based targeted prodrug, which is activated by PSMA-mediated cleavage of an inert masking peptide. Similar to TG, it is an inhibitor of the calcium adenosine triphosphatase (SERCA) pump protein that is necessary for cellular viability. Mipsagargin was used in patients with advanced solid tumours and established a recommended phase II dosing (RP2D) regimen. It was found to have an acceptable tolerability and favourable pharmacokinetic profile in patients with solid tumours [162].

## 17. Bruceantin

**Figure 21:**
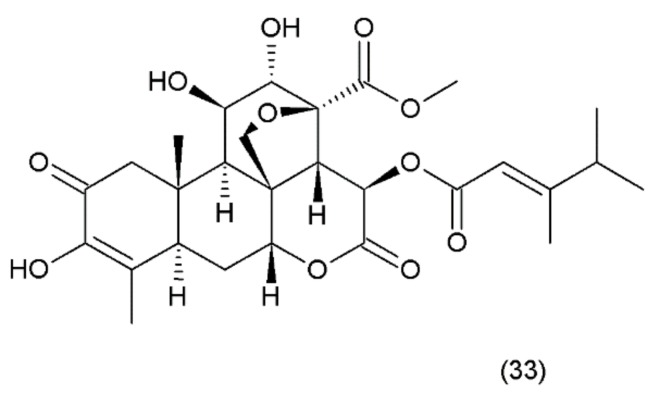
(**33**) Bruceantin.

Bruceantin (33) (Figure 21) was first isolated from *Brucea antidysenterica* (Simaroubaceae), a tree that grows in Ethiopia, which is used by the local population in the treatment of “cancer” [142].

Brucantin was synthesized by Sasaki and Murae (1989) [163]. The anticancer activity of bruceantin has been observed in the treatment of various cancers, such as B16 melanoma, colon 38, and L1210 and P388 leukaemia, in mice. The activity of bruceantin has been studied with several leukaemia, lymphoma, and myeloma cell lines. However, no objective tumour regressions were observed in clinical development in phase I and II clinical trials, so application of this drug was terminated. On the other hand, resumed research exhibited the activity of bruceantin against leukaemia, lymphoma, and myeloma cell lines in animal models with advanced stages of the disease. It was demonstrated that treatment of HL-60 and RPMI 8226 cell lines induced apoptosis, and this involved the caspase and mitochondrial pathways. Moreover, an in vivo study using RPMI 8226 human-SCID xenografts revealed that bruceantin induced regression in early as well as advanced tumours. The main mechanism of bruceantin action associated with anticancer activity is the inhibition of protein synthesis through interaction with peptidyltransferase, which blocks the formation of peptide binding [164]. It was demonstrated that the alcohol extract of the fruit of *Brucea javanica* (Fructus Bruceae) possesses significant cytotoxicity in pancreatic adenocarcinoma cell lines. Seven quassinoids were identified after their fractionation and purification, including brusatol, bruceine D, bruceine H, yadanzioside A, yadanzioside G, javanicoside C, and bruceantinoside A. Brusatol exhibited the most potent in vitro antipancreatic cancer action on PANC- 1 and SW1990 cell lines [165].

## 18. Marine Natural Compounds

### 18.1. Psammaplin

**Figure 22:**
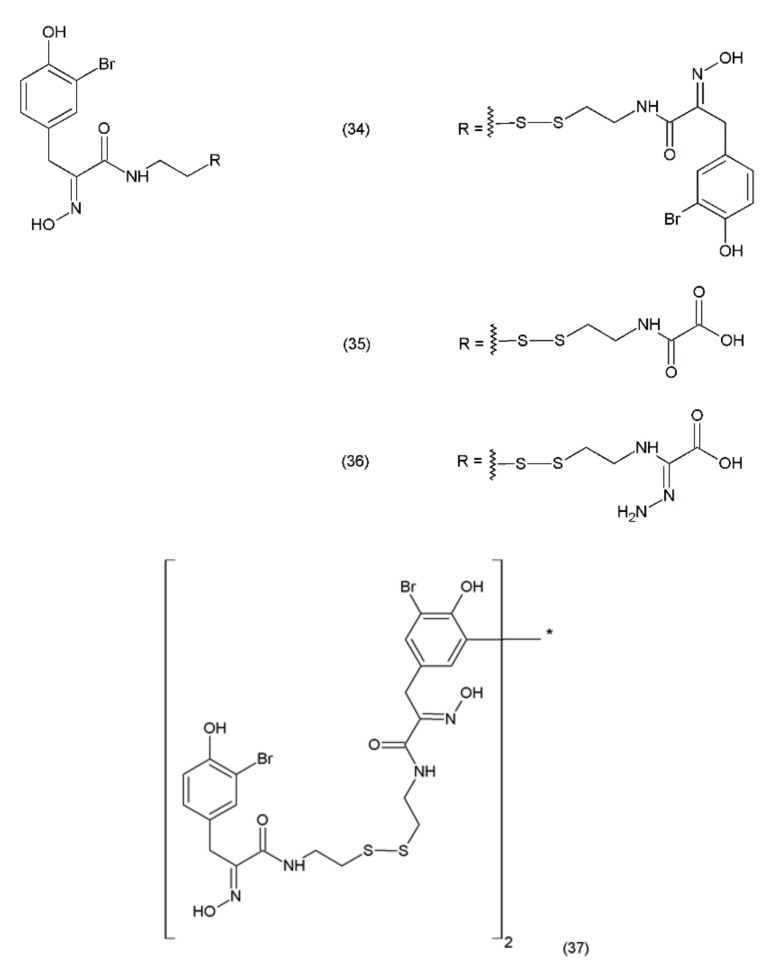
Psammaplin derivatives: (**34**) psammaplin A, (**35**) psammaplin F, (**36**) psammaplin G, (**37**) biprasin.

Psammaplin A (PsA) (34) is an anticancer compound isolated from *Poecillastra sp.* and *Jaspis sp.* It was first isolated from *Psammaplin aplysilla* sea sponges [169]. PsA and biprasin are present in marine microalgae, cyanobacteria, and in the heterotrophic bacteria living in association with invertebrates (e.g., sponges, tunicates, and soft corals). Psammaplin A is a phenolic compound containing a disulphide bridge; it occurs in nature in the form of monomers or dimers. Additionally, psammaplin A contains a bromotyrosine ring. PsA exhibits varying effects on different pathways. Psammaplin A was synthesized by Hoshino and co-workers (1992) [170]. This drug exhibits antitumour properties and inhibits aminopeptidase N, which is a key factor in tumour cell invasion and angiogenesis [171]. Psammaplin A has been demonstrated to inhibit the proliferation of leukaemia cells through the induction of apoptosis, as well as the cell growth of Bap1-null cells, while causing minimal toxicity to human neuroblastomal SKN cells [172,173]. The results obtained are particularly strong when this drug is used in combination with camptothecin (a DNA damage inducing drug). This indicates that psammaplin A could serve as a potential adjuvant therapy for the cancer patients, particularly for Bap1-null lung cancer patients who are treated with the DNA damage-inducing therapies [173]. Psammaplin A (34) and its derivatives, psammaplin F (35), psammaplin G (36), and biprasin (37) (Figure 22), inhibit histone deacetylase (HDAC) activity, which performs a key role in tumourigenesis and angiogenesis. 

**Figure 23:**
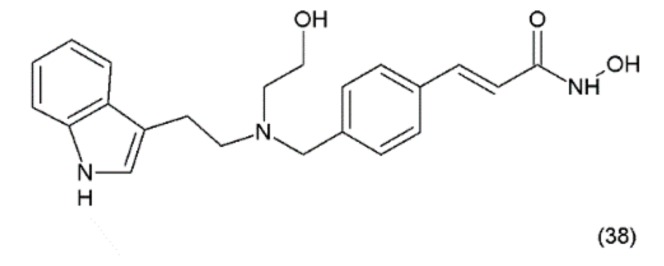
(**38**) NVP-LAQ824.

Psammaplin A also inhibits various enzymes, including topoisomerase II, farnesyl-protein transferase, leucine aminopeptidase, and chitinase [174]. Additionally, this compound causes cell-cycle arrest and the induction of apoptosis in numerous human cancer cells. PsA has demonstrated antiproliferative activity and induces cell cycle arrest or apoptosis in Ishikawa human endometrial cancer cells [175]. Furthermore, psammaplin F is a selective histone deacetylase inhibitor, while psammaplin G is a selective DNA methyltransferase inhibitor. NVP-LAQ824 (38) (Figure 23) is a synthetic derivative of indol and a strong histone deacetylase inhibitor that is used in the treatment of solid and haematological malignancies. NVP-LAQ824 exhibits antileukaemic activity when administered in the low nanomolar range. It has also been used in clinical trials in patients with solid tumours or leukaemia. NVP-LAQ824 demonstrated low host cell toxicity in HCT116 colon cancer and A549 human lung cancer xenografts; also, it caused apoptosis in the A549 lung cancer cell line and the normal human fibroblast cell line [176].

### 18.2. Didemnin B

**Figure 24:**
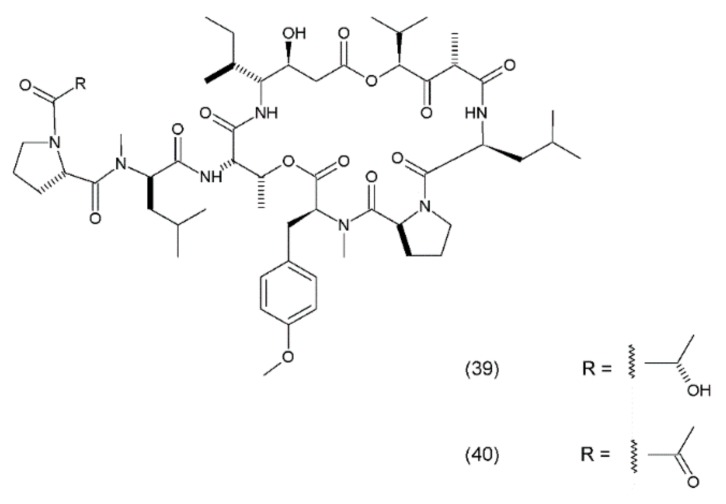
(**39**) Didemnin B, (**40**) plitidepsin.

Didemnin B (**39**) is a cyclic depsipeptide which was isolated from the marine tunicate *Trididemnum solidum*. It was the first marine natural compound to enter clinical trials as an antitumour agent. It exhibits structural similarity to the cyanobacterial metabolites [177]. Didemnin B was synthesized by Ramanjulu and co-workers (1997) [178]. In clinical trials, it exhibited anticancer activity against a variety of tumours, such as bronchial carcinoid, colon cancer, oesophageal cancer, malignant melanoma, medullar thyroid carcinoma (MTC), metastatic breast cancer, non-small cell lung cancer, renal cancer, and squamous cell cervical cancer [179]. Didemnin B inhibits the synthesis of ribonucleic acid (RNA), DNA, and proteins and binds non-competitively to palmitoyl-protein thioesterase [180]. It has been demonstrated that didemnin B is able to induce apoptosis in a wide range of transformed cell lines. Didemnin B was shown to induce apoptosis in normal lymphocytes only after mitogenic stimulation [181]. The use of this drug in leukaemia requires further research. Furthermore, rapamycin inhibits didemnin-induced apoptosis in human HL60 cells, suggesting the activation of the FK506-binding protein apoptotic pathway [182]. Didemnin B (Figure 24) possibly modulates the activity of FK506-binding proteins as a part of its immunomodulatory process, and thus leads to cell death via apoptosis. Plitidepsin (aplidine) (40) is an anticancer agent obtained from the ascidian *Aplidium albicans*. It binds specifically to the alpha subunit of the eukaryotic Elongation Factor 1 (eEF1A2), resulting in tumour cell death via apoptosis. 

eEF1A2 is overexpressed in human tumours, favouring tumour cell proliferation and inhibiting apoptosis. Previous experiments have demonstrated that plitidepsin localises in tumour cells that are sufficiently close to the eEF1A2, indicating the formation of drug–protein complexes in the living cells [183]. Plitidepsin also interferes with the synthesis of DNA and proteins and induces cell cycle arrest in the G1 and G2 phases of the cell cycle [184]. This compound is currently under clinical development for haematological cancers, including a phase Ib trial of a triple-drug combination of plitidepsin, bortezomib, and dexamethasone for relapsed or refractory multiple myeloma and a phase II study for relapsed or refractory angioimmunoblastic T-cell lymphoma. In a study assessing the clinical benefit of plitidepsin in patients with advanced medullary thyroid carcinoma (MTC), plitidepsin appeared to be able to induce clinical benefits in patients with pretreated MTC, and its toxicity was manageable at the recommended dose [185]. Additionally, aplidine appears to work more selectively against leukaemia and lymphoma cells than against normal cells. Furthermore, the activity of aplidine has been observed to be independent of the other anticancer drugs that are commonly used in the treatment of leukaemia and lymphoma, suggesting that aplidine may be effective in cases which have proven unresponsive to the other anticancer agents/drugs [186].

### 18.3. Dolastatin

**Figure 25:**
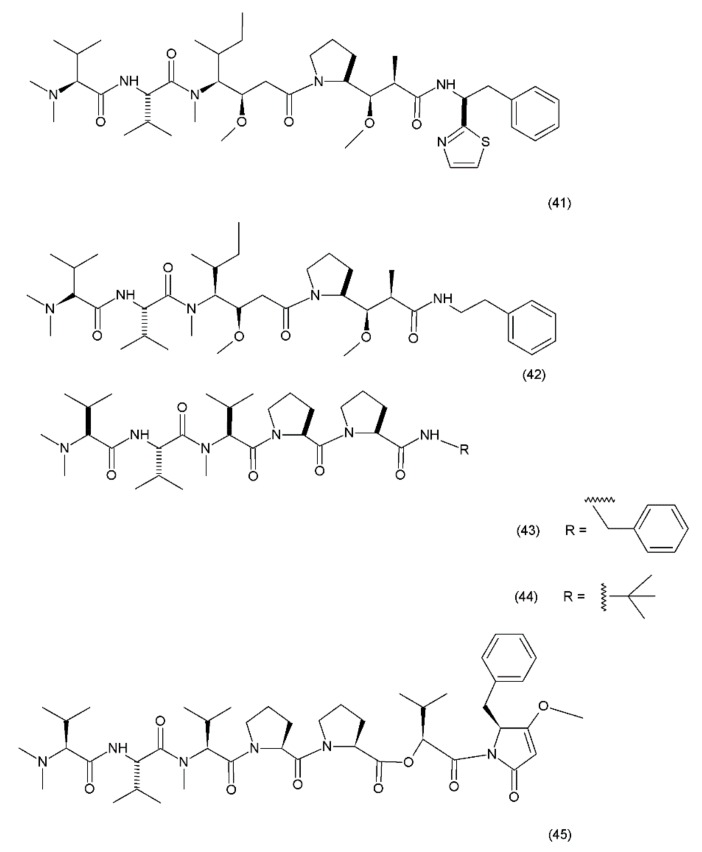
Dolastin 10 and dolastin 15 derivatives: (**41**) dolastatin 10, (**42**) auristatin PE, (**43**) cematodin, (**44**) synthadotin, (**45**) dolastatin 15.

Dolastatin 10 (**41**) (Figure 25) was first isolated in 1972 from the sea hare *Dolabella auricularia*. However, the performance of this drug was very low due to its low concentration. Significantly higher yields were obtained from the marine cyanobacteria *Symploca hydnoides* and *Lyngbya majuscule* which were also synthezised by Tomioka and coworkers (1991) [187].

Dolastatin 10 is a pentapeptide with four of its amino acid residues being structurally unique (dolavaline, dolaisoleucine, dolaproline, and dolaphenine, in addition to valine). This compound has demonstrated pronounced antineoplastic activity. Interestingly, at the time of its discovery, it was referred to as the most potent antiproliferative agent. The semisynthetic/synthetic derivatives of dolastatin, such as auristatin PE (TZT–1027, soblidotin) (**42**), synthadotin (ILX651) (**43**), and cematodin (LU–103793) (**44**), which usually possess improved pharmacological and pharmacokinetic properties [188,189]. The antitumour activity of TZT–1027 (soblidotin), a synthetic derivative of dolastatin 10, was observed to be superior to that of the existing anticancer drugs, such as paclitaxel and vincristine; TZT–1027 is currently undergoing phase I trials for the treatment of solid tumours [189]. The third-generation dolastatin 15 (**45**) derivative, synthadotin (ILX–651, tasidotin), is another antitumour agent that is currently undergoing phase II trials after its successful use in the phase I trials [188,190]. Dolastatin 10 has been used in trials for the treatment of sarcoma, leukaemia, lymphoma, liver cancer, kidney cancer, etc. Although dolastatin has not been directly used clinically for the treatment of moderate peripheral neuropathy in 40% of the patients, its derivatives, such as soblidotin and auristatin PE, were designed with the goal of maintaining the antitumour activity potential while reducing the toxicity of the parent compound [191]. Auristatin is the semisynthetic derivative of dolastatin 10; to produce this compound, the thiazole ring was removed from the original dolaphenine residue. The application of auristatin led to the inhibition of growth in the P388 leukaemia cells and the diminution of three solid-tumour cell lines (colon-26 adenocarcinoma, B16 melanoma, and M5076 sarcoma) with an efficacy equivalent to or greater than that of dolastatin 10 [176]. Furthermore, TZT–1027 was shown to be effective in two human xenograph models—breast carcinoma MX–1 and lung carcinoma LX–1 [192]. Dolastatin 15, which was synthesized by Patino et al. (1992) [193], is a small linear depsipeptide compound which is considered a promising anticancer drug that exhibits potency against breast and liver cancers, solid tumours, and certain types of leukaemia. In contrast to dolastatin 10, dolastatin 15 binds directly to the vinca domain of tubulin, and its mechanism of action is similar to that of the vinca alkaloids. It inhibits the microtubule assembly by interfering with tubulin formation, thereby disrupting mitotic cell division and consequently, inducing apoptosis in cancer cells. Synthadotin (ILX651) is an orally-active, third-generation synthetic derivative of dolastatin 15 that contains a terminal tert-butyl moiety. Synthadotin has been used in three phase II clinical trials in patients with locally-advanced or metastatic non-small cell lung cancer, and in patients with hormone-refractory prostate cancer who have been treated previously with docetaxel [194,195]. Synthadotin has also been demonstrated to be a well-tolerated drug for the treatment of patients with inoperable locally-advanced or metastatic melanoma [194].

### 18.4. Ecteinascidin

**Figure 26:**
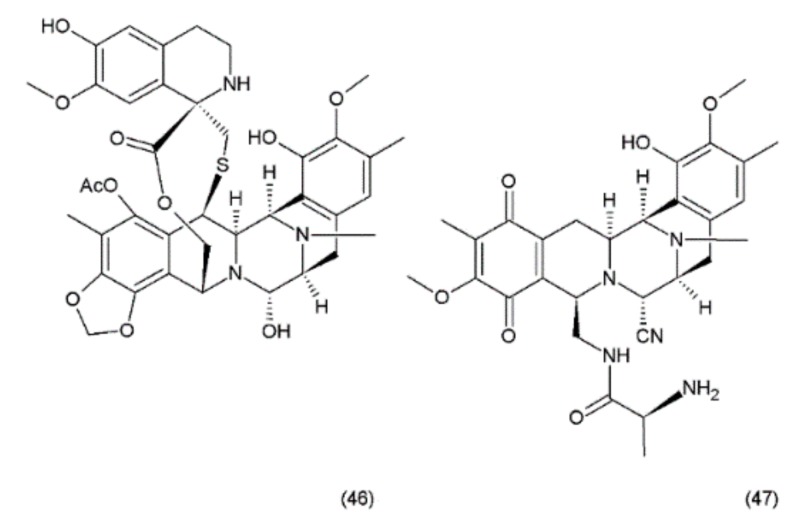
(**46**) Ecteinascidin, (**47**) cyanosafracin B.

Ecteinascidin–743 (ET–743, Trabectedin) (**46**) is a tetrahydroisoquinoline alkaloid isolated from the Caribbean marine tunicate *Ecteinascidia turbinata*. This compound contains three fused tetrahydroisoquinoline rings. Trabectedin has previously been isolated from aquacultures and even produced synthetically, eventually leading to the semisynthesis of ET–743 (**49**) from cyanosafracin B (**50**). In turn, cyanosafracin B could be obtained from the fermentation of the marine bacterium *Pseudomonas fluorescens* [196] (Figure 26). 

Although less potent in vivo than its N-demethyl analogue (ET–729), trabectedin exhibits highly potent antitumour activity with a cytotoxicity (IC_50_) value of 0.5 ng/mL against the L1210 leukaemia cells [176]. Trabectedin interacts with DNA through the covalent interactions of two of its tetrahydroisoquinoline rings with the minor groove of the DNA double helix, while the third ring protrudes from the DNA duplex, apparently allowing interactions with the adjacent nuclear proteins [197,198]. The interactions of trabectedin with DNA trigger a cascade of events that are associated with several transcription factors such as the DNA-binding proteins and with DNA-repair pathways, making this drug different from the other DNA-interacting agents. Additionally, this compound leads to modulation of the production of cytokines and chemokines by tumour and normal cells. It has been suggested that the anticancer activity of trabectedin could also be associated with changes in the tumour microenvironment [198]. Trabectedin treatment demonstrated the greatest response in advanced ovarian, breast, and mesenchymal tumours which had been pretreated heavily with platinum/taxane therapy. Phase II clinical trials of this drug were conducted for ovarian, endometrial, breast, prostate, and non-small cell lung cancers, with notable recent success in the combination drug therapy [195]. Promising combinations of trabectedin with other anticancer drugs were observed during preclinical studies. One of these combinations (trabectedin–PEGylated liposomal doxorubicin) was recently authorised by the European Commission for the treatment of patients with relapsed platinum-sensitive ovarian cancer [198].

### 18.5. Halichondrin B

**Figure 27:**
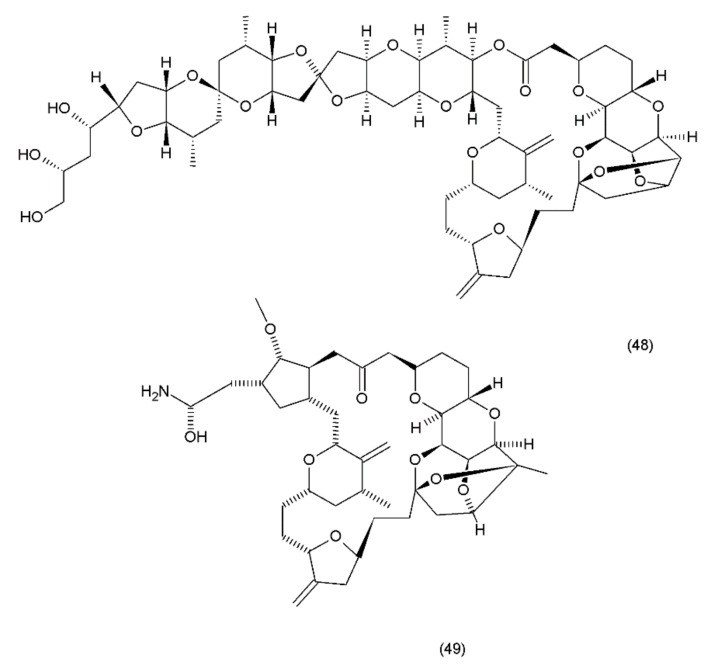
(**48**) Halichondrin B, (**49**) halichondrin analog E7389.

Halichondrin B (48) is a complex polyether that is isolated from marine animals such as sponges and tunicates. Halichondrins were first isolated from the Japanese sponge *Halichondria okadai* by Uemura et al. (1985) [199], and their structures were determined using X-ray crystallography. Subsequently, halichondrin B and several of its natural analogues (49) (Figure 27) were isolated from various unrelated sponges, including the *Lissodendoryx sp.*, *Phakellia carteri*, and *Axinella sp*. However, they may also be obtained from several other species, especially fish [172]. Halichondrin B, as well as palytoxin and maitotoxin, exhibit structural complexity and were synthesized by Aicher et al. (1991) [200]. Several studies have examined their mechanisms for cell toxicity, and it was discovered that the halichondrins are potent tubulin inhibitors that bind non-competitively to the vinca-binding site and cause a characteristic G2–M cell-cycle arrest through a concomitant disruption of the mitotic spindle. Exquisite anticancer activity of halichondrin B and homohalichondrin B against the murine cancer cells was reported in both culture and in vivo studies. These studies confirmed that both compounds are highly cytotoxic (IC_50_ values of 0.3 nM and 1 nM, respectively, against the L1210 murine leukaemia cells), causing the accumulation of cells arrested in mitosis at toxic concentrations. The studies also confirmed that both compounds inhibit the polymerisation of purified tubulin and microtubule assembly that is dependent on microtubule-associated proteins [201].

## 19. Conclusions

Modern chemotherapy utilises many substances of plant and aquatic origin. These compounds have cytotoxic properties with many different mechanisms of action, such as the inhibition of tumour cell growth, the induction of apoptosis, DNA damage, the inhibition of topoisomerases I and II, the induction of apoptosis, and others. Studies have shown that plant-derived compounds in combination with anticancer drugs have great potential to destroy tumour cells while not affecting normal cells such as lymphocytes and fibroblasts. Several of these substances are undergoing preclinical testing or are in the subsequent stages of clinical trials. Although these substances exhibit high potential for cancer treatment, they also exhibit several side effects. Certain substances are not suitable for use as drugs due to their high toxicity, although their synthetic/semisynthetic derivatives have been used as drugs in cancer treatment. The side effects of anticancer drugs may be reduced by using nanoparticle encapsulations to transport the drugs to their target sites. Drugs may also be administered in the form of liposomes, which serve as carriers for the drug. An additional argument associated with the nanoparticles is the limitation of multi-drug resistance. Although several compounds of plant origin are known to serve as anticancer drugs, modern medicine lacks effective drugs against certain types of cancers, for example, metastatic pancreatic adenocarcinoma and castration-resistant prostate cancer. The marine environment is characterized by a huge diversity of organisms, creating a wide range of opportunities for acquiring new substances with anticancer activity, which is a challenge for scientists involved in this research. We also need to improve techniques related to the isolation of substances from natural materials to obtain higher yields with concern for the environment. Considering the huge costs associated with the discovery and development of effective anticancer drugs, natural compounds can be an inexhaustible source here.

## Figures and Tables

**Table 1:** Anticancer effects of natural compounds from plants in different experimental systems.

Natural Compounds	Origin	Cell Line	Dose	Mechanisms of Action	References
*Catharanthus* alkaloids	*Catharanthus roseus* Pink	acute lymphocytic leukaemia, non-small cell lung cancer, bladder cancer	<1 μmol >1–2 μmol	inhibit the microtubule dynamics and stabilise them disintegrate the microtubules and damage the mitotic spindle, leading to the inhibition of mitosis and causing apoptosis	[17,28]
*Viscum Album* extract	*Viscum album*—Mistletoe plant *Viscum album* that are used in therapy are usually a mixture of the extracts obtained from various host trees (oak, apple, pine, fir, willow, birch, lime, etc.).	human colon cancer cells (Colo 320 HSR) breast cancer cells (MFM-223, HCC–1937, KPL-1, MCF-7)	10–100 ng/mL IC_50_: VAP-Qu 0.05–0.11 mg/mL VAP-M 0.10–0.12 mg/mL VAP-A 0.07–0.31 mg/mL VAP-P 1.61–2.14 mg/mL MLI 38–410 ng/mL	activation of the intrinsic (activated Caspase–2 and 9) extrinsic (activated Caspase–2, 3 and 8) pathways of apoptosis activation of only the mitochondrial pathway of apoptosis early cell-cycle inhibition followed by apoptosis activation of the mitochondrial pathway of apoptosis	[29,30,31,32,33,34,35,36,37]
Taxanes	*Taxus baccata* - European yew *Taxus brevifolia* - the Pacific yew	PTX: breast cancer cells (MCF-7), lung adenocarcinoma (A549), cervical carcinoma (HeLa), grade III astrocytoma (U373), colon adenocarcinoma (HT-29), adenocarcinoma (OVG-1), pancreatic adenocarcinomas (PC-Sh) DTX: human lung cancer (A549)	IC_50_ of PTX: HeLa 2.6 nM A549 4.1 nM U373 4.2 nM MCF-7 2.5 nM HT-29 2.8 nM OVG-1 4.0 nM PC-Sh 7.5 nM IC_50_ of DTX: A549 4.26 + 0.51 nM	mitosis inhibitors affect the microtubules	[38,39,40,41,42,43,44,45,46,47,48,49,50,51,52,53,54,166,167]
Camptothecin	*Camptotheca acuminata* Decne (Nyssaceae)	human colon HCT116, breast cancer cells (MCF-7), prostate cancer (DU145), leukaemia (CEM)	IC_50_ of CPT: HCT116 169.5 nmol/L MCF-7 180.3 nmol/L DU145 15,700 nmol/L CEM 13,600 nmol/L	binds to a complex consisting of DNA and topoisomerase I stabilises the topoisomerase I (TOP1) complex and the ruptured DNA	[55,56,57,58,59,60,61,62,63,64,65,66,67,68,69,70,71,72,73,74,75,76,77,78,79,80,81]
Combretastatin	*Combretum caffrum* - “African willow”	human thyroid papillary carcinoma cell (TPC1)	>5 µM	binds to β-tubulin at what is known as the colchicine site causes the destabilisation of the microtubules	[83,84,85,86,87,88]
Podophyllotoxins	*Podophyllum peltatum, Podophyllum emodi* *Podophyllum versipelle, Linum* *Juniperus*	small-cell lung cancer (SCLC)	>1 µg/mL (etoposide)	disrupt the organisation of the karyokinetic spindle single-strand and double-strand breaks in DNA through their interactions with DNA topoisomerase II induce cell cycle arrest in the G2-phase of the cell cycle	[89,90,91,92,93,94,95,96,97,98,99,100]
Geniposide and its derivatives	*Gardenia jasminoides* Ellis—Rubiaceae	human non–small-cell lung cancer H1299 cell	IC_50_ of genipin 351.5 μM	activation of the mitochondrial execution pathway by Caspase-9 and -3 increase levels of Bax in response to p38MAPK signalling initiation of the mitochondrial death cascade	[101,102,103,104,105,106,107,108]
Colchicine	*Colchicum* genus (e.g., the autumn crocus, *Colchicum autumnale*, also known as “meadow saffron”)	hepatocellular carcinoma HepG2	10 μM	depolymerises the microtubules at high concentrations stabilizes the microtubule dynamics at low concentrations limits mitochondrial metabolism in cancer cells	[109,110,111,112,113,114]
Artesunate	*Artemisia annua L.* medicinal—Asteraceae	chronic myeloid leukaemia K562 cells	2 μM	antiangiogenic effect inhibits VEGF expression	[115,116,117,118,119,120,121,122,123,124,125,126]
Homoharrigtonine	*Cephalotaxus*—Cephalotaxaceae (*C. harringtonia* K. Koch, *C. haianensis qinensis*)	gallbladder adenocarcinoma cell line (Mz-ChA-1), colorectal adenocarcinoma cell line (HT-29)	Mz-ChA-1 0.1 μM HT-29 1 μM	blocks synthesis in the peptidyl transferase centre	[127,128,129,168]
Salvicine	*Salvia pronitis* Hance—Labiatae	leukaemia cell (P388, HL-60), stomach cancer cell (SGC-7901)	IC_50_: P388 3.49 μM HL-60 3.57 μM SGC-7901 1.84 μM	breaks two strands of DNA by facilitating TOP2 activity inhibits re-ligation	[130,131,132]
Elipticine	*Ochrosia elliptica* Labill	leukaemia (HL-60, CCRF-CEM) cells	IC_50_: HL-60 0.64 μM CCRF-CEM 4.7 μM	disrupts the cell-cycle by regulating the expression of some kinases (cyclin B1 and Cdc2) generates free radicals	[133,134,135,136,137,138]
Roscovitine	*Raphanus sativus* L.—Brassicaceae	highly metastatic and invasive breast cancer cells MDA-MB231	10 μg/mL	inhibits cyclin-dependent kinase (CDK) activity, which preferentially inhibits numerous target enzymes such as CDK1, CDK2 and CDK5 leading to cell-cycle arrest in the G1 and G2 phases	[139,140,141,142,143,144,145,146,147,148]
Maytansin	*Maytenus serrata*—Celastracea, *Maytenus ovatus*	COLO 205 cells	IC_50_: 0.08 nM	inhibits microtubule assembly by binding to tubulin	[149,150,151,152,153,154,155]
Tapsigargin	*Thapsia garganica L.*—Apiaceae	mouse embryonic fibroblast (MEF) cells	3 μM	inhibits sarcoplasmic/endoplasmic reticulum calcium adenosine triphosphatase (SERCA) in the sarco/endoplasmic reticulum, raises the intracellular calcium concentration, enhances the endoplasmic reticulum stress, caspase activation, releases apoptotic factors from the mitochondria and directly activates the calcium-dependent endonucleases, inhibits the fusion of autophagosomes with lysosomes	[156,157,158,159,160,161,162]
Bruceantin	*Brucea antidysenterica*—Simaroubacea, *Brucea javanica*—Fructus Bruceae	human pancreatic cancer cells (PANC-1, SW1990)	IC_50_ of Bruceantinoside A: PANC-1 16.90 μM SW1990 14.08 μM	inhibits protein synthesis through interaction with peptidyltransferase, which blocks the formation of peptide binding	[163,164,165]

IC_50_—The concentration corresponding to a survival rate of 50% is defined as the IC_50_.

**Table 2:** Anticancer effects of marine natural compounds in different experimental systems.

Natural Compounds	Origin	Target Cells	Dose	Mechanisms of Action	References
Psammaplin	*Poecillastra sp.*, *Jaspis sp., Psammaplin aplysilla* marine microalgae, cyanobacteria, heterotrophic bacteria living in association with the invertebrates (e.g., sponges, tunicates, and soft corals)	human endometrial Ishikawa cancer cells	5 μg/mL	antiproliferative effects, selectively induces genes related to cell-cycle arrest and apoptosis inhibits the activity histone deacetylase (HDAC), chitinase topoisomerase II, farnesyl-protein transferase, leucine aminopeptidase,	[169,170,171,172,173,174,175,176]
Didemnin	*Trididemnum solidum,* *Aplidium albicans*	MOLT-4 cells (human T lymphoblast; acute lymphoblastic leukaemia)	5–30 nM	cell-cycle phase perturbations, inhibits the synthesis of RNA, DNA, and proteins	[177,178,179,180,181,182,183,184,185,186]
Dolastatine	*Dolabella auricularia,* *Symploca hydnoides, Lyngbya majuscula*	human breast cancer cells (MCF-7, R-27)	20 ng/mL	disrupts mitotic cell division	[187,188,189,190,191,192,193,194,195]
Ecteinascidin	*Ecteinascidia turbinata,* *Pseudomonas fluorescens*	L1210 leukaemia cells	IC_50_ 0.5 ng/mL	binds in the minor groove of DNA to induce an unprecedented bend in the DNA helix towards the major groove interferes with cellular transcription-coupled nucleotide excision repair to induce cell death and cytotoxicity	[196,197,198,199,200]
Halichondrin B	*Halichondria okadai,* *Lissodendoryx sp.,* *Phakellia carteri,* *Axinella sp.*	L1210 murine leukaemia cells	IC_50_ 0.3 nM	tubulin inhibitor G2–M cell-cycle arrest	[199,200,201]

IC_50_—The concentration corresponding to a survival rate of 50% is defined as the IC_50_.

## Abbreviations

ATC	Anaplastic thyroid cancer
CA4P	Combretastatin A-4 phosphate
CDK	Cyclin-dependent kinase
CRPC	Castration-resistant prostate cancer
mCRPC	Metastatic castration-resistant prostate cancer
CTX	Cabazitaxel
DNA	Deoxyribonucleic acid
DPT	Deoxypodophyllotoxin
DTX	Docetaxel
GS	Geniposide
HDAC	Histone deacetylase
IC_50_	The concentration corresponding to a survival rate of 50%
LNCaP	Prostate cancer cell line
MBC	Metastatic breast cancer
MDR	Multi-drug resistance
MM	Multiple myeloma
MTC	Medullary thyroid carcinoma
NSCLC	Non-small cell lung cancer
PEDF	Pigment epithelium-derived factor
PI3K	Phosphatidylinositol-3-kinase
PPT	Podophyllotoxin
PsA	Psammaplin A
PSMA	Prostate-specific membrane antigen
PTX	Paclitaxel
RNA	Ribonucleic acid
RP2D	Recommended phase II dosing
SERCA	Sarcoplasmic/endoplasmic reticulum calcium adenosine triphosphatase
SCLC	Small cell lung cancer
TG	Tapsigargin
TOP1	Topoisomerase I
TPA	12-O-tetradecanoylphorbol-13-acetate
TPC1	Thyroid papillary carcinoma cell line
VAs	Vinca alkaloids
VAE	*Viscum album* extract
VEGF	Vascular endothelial growth factor
VT	Viscotoxins
